# Chromosome-Scale Genome and Transcriptomic Analyses Reveal Differential Regulation of Terpenoid Secondary Metabolites in *Hericium coralloides*

**DOI:** 10.3390/jof10100704

**Published:** 2024-10-09

**Authors:** Kexin Meng, Junyi Lv, Tuo Zhang, Yuanyuan Liu, Peng Zhang, Yue Zhang, Banghui Hu, Qianhui Huang, Baogui Xie, Junsheng Fu

**Affiliations:** 1 College of Life Sciences, Fujian Agriculture and Forestry University, Fuzhou 350002, China; mkx15736938616@163.com (K.M.); lovesicklqf@outlook.com (J.L.); zhangtuo_1001@163.com (T.Z.); lyylyy0815@163.com (Y.L.); zp1960390216@163.com (P.Z.); great_zhangyue@163.com (Y.Z.); hbh1918210324@163.com (B.H.); 2 Mycological Research Center, Fujian Agriculture and Forestry University, Fuzhou 350002, China; 3 College of Biological Sciences and Engineering, Ningde Normal University, Ningde 352100, China

**Keywords:** *Hericium coralloides*, genome and transcriptome, terpenoids, cluster gene

## Abstract

Construction of the genome of *Hericium coralloides*, a species of edible mushroom, and identification of the genes involved in terpenoid biosynthesis can determine the biology and genetics of terpenoids. The present study describes the assembly of a high-quality chromosome-scale genome of *H. coralloides* using Pacbio HiFi sequencing and Hi-C technology. This genome consisted of 13 chromosomes, a total size of 43.6 Mb, contigs of N50 3.6 Mb, GC content at 54%, and BUSCOs integrity of 96.9%. Genes associated with terpenoid biosynthesis were predicted by KEGG enrichment analysis and homologous alignment. The *Her011461* and *Her008335* genes, encoding proteins in the terpenoid backbone synthesis pathway, were found to encode geranylgeranyl pyrophosphate and farnesyl diphosphate synthases, key enzymes in the biosynthesis of geranylgeranyl diphosphate, a precursor of several diterpenoids. *Her011463* was found to be involved in regulating diterpene cyclase. The *Her005433*, *Her006724*, *Her010605*, and *Her010608* genes were found to encode sesquiterpene synthesis. Most of these genes were more highly expressed in dikaryotic mycelia than in the primordium and fruiting bodies, indicating that terpenoids may be more abundant in dikaryotic mycelia. To our knowledge, this study is the first to assemble the *H. coralloides* genome at the chromosome scale and to identify the genes involved in terpenoid biosynthesis.

## 1. Introduction

*Hericium coralloides* is a species of edible and medicinal mushroom, of Division Basidiomycota, Class Agaricomycetes, Order Russulales, and Family Hericiaceae [1]. The chemical composition of *H. coralloides* is complex and includes many small molecules, such as erinacines, phenols, steroids, and pyranone substances. Erinacines are diterpenoid compounds with a cyathane backbone and various biological activities, such as the activation of NGF synthesis and inhibition of Helicobacter pylori, as well as antioxidant, anti-inflammatory, and anti-tumor properties [2,3,4,5]. *H. coralloides* is widely used in foods, cosmetics, pharmaceuticals, and other products. Few studies to date, however, have evaluated the active substances of *H. coralloides*, thus greatly limiting its utilization as a resource. Construction of the complete genome of *H. coralloides* may provide genetic information that can help distinguish the desired traits and apply them to industrial production.

Although the *H. coralloides* (tvtc0002) genome has been published in the National Center of Biotechnology Information (NCBI) database, this genome has not yet been assembled at the chromosome scale [6]. Only 307 contigs were assembled at the contig scale. The rapid development of single-molecule high-fidelity (HiFi) long-read sequencing technology and high-throughput chromosome conformation capture sequencing (Hi-C) technology has enabled the determination of high quality chromosome-level genomes of many model species [7,8,9], including edible fungi such as *Peurotus giganteus* [10], *Phellinus gilvus* [11], *Hericium erinaceus* [12], *Russula griseocarnosa* [13], *Stropharia rugosoannulata* [14], and *Lentinus edodes* [15]. To facilitate genome sequencing, analysis, and annotation, genomic DNA was extracted from mononuclear mycelia regenerated from *H. coralloides* protoplasts, with the genome assembled using HiFi and Hi-C techniques.

*H. coralloides* genes related to the biosynthesis of terpenoids were identified by KEGG enrichment analysis of differentially expressed genes in the fungal transcriptome. The levels of expression of differentially expressed genes in the terpenoid backbone synthesis pathway were analyzed at different growth stages, with the genes affecting the synthesis of diterpenoids and sesquiterpenes identified by BLAST homology alignment. In addition, gene clusters involved in terpenoid synthesis were predicted using antiSMASH 6.0.1software. Overall, the results of this study will provide fundamental information on terpenoid biosynthesis pathways and the production of terpenoids in *H. coralloides* and provide multi-omics insights into the biosynthesis of terpenoids.

## 2. Materials and Methods

### 2.1. Materials

**Figure 1:**
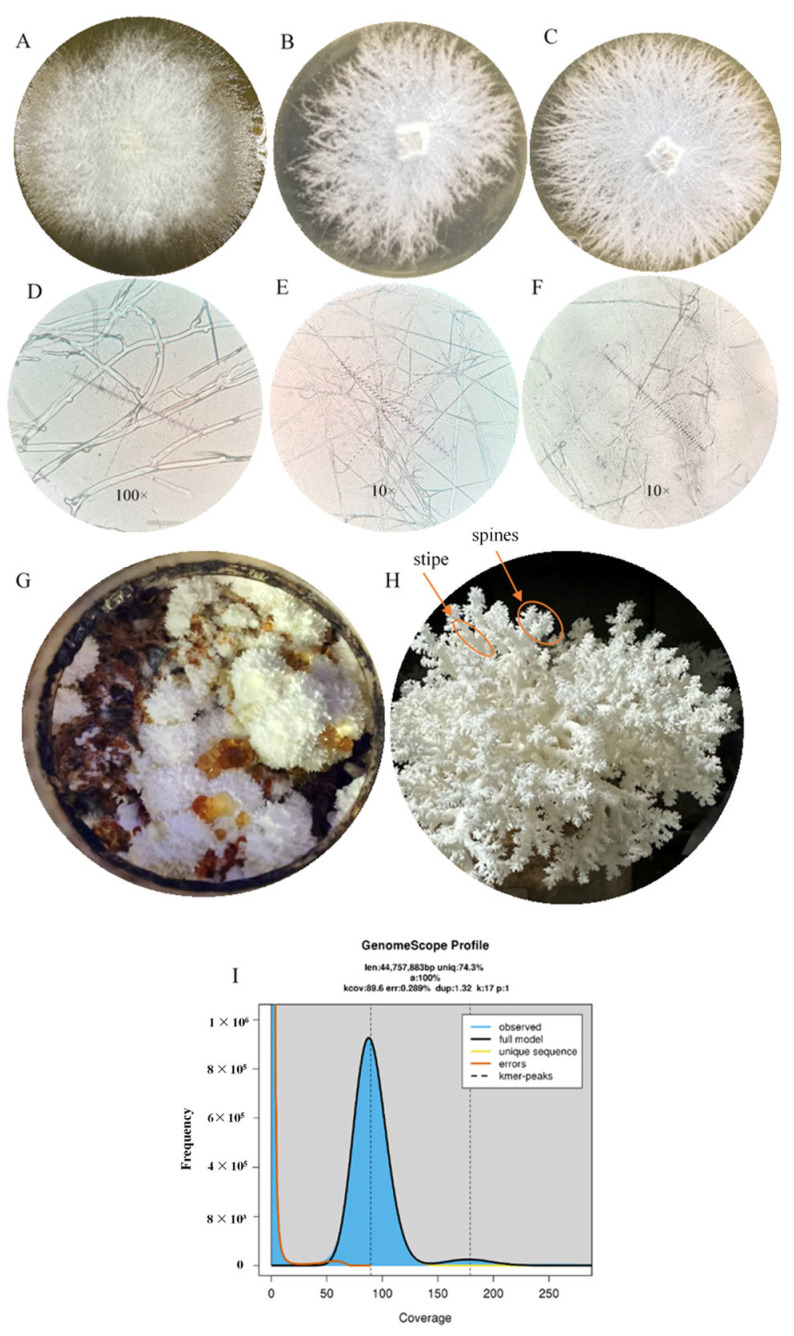
Phenotype and microscopic morphology of (**A**–**C**) Morphology of the DK, MK-1, and MK-2 strains grown on potato dextrose agar (PDA). (**D**) Mycelial morphology of DK at 100× magnification. (**E**,**F**) Mycelial morphology of MK-1 and MK-2 at 10× magnification. (**G**) The primordium of DK (FB-A). (**H**) Mature fruiting bodies (FB) of DK; the yellow arrows indicate the stipe (FB-B) and the spines (FB-C). (**I**) Genome K-mer distribution map.

A dikaryon strain of *H. coralloides* named ‘77’ was provided by the Fujian Edible Fungi Germplasm Resource Collection Center of China. This strain was cultured on potato glucose agar (PDA) in the dark at 25 °C for 2 weeks (Figure 1A); lysozyme was purchased from the Guangdong Institute of Microbiology (Guangdong Province, China). RNA extraction kits were from Omega Bio-tek, Inc (Guangdong Province, China). TransScript All-in-one First-Strand cDNA Synthesis SuperMix for qPCR (One Step gDNA Removal) reverse transcription kits and PerfectStart Green qPCRSuperMix quantitative kits were purchased from Beijing Quanshijin Biotechnology Co., Ltd. (Beijing Province, China). All chemicals, including mercaptoethanol and anhydrous ethanol, were of analytical grade.

### 2.2. Preparation of Protoplasts

Fungi were treated with 2% lysozyme enzyme solution to enzymolyze mycelia, and the concentration of protoplasts was determined on a hemocytometer (Shanghai Qiujing Biochemical Reagent Instrument Co., Ltd., Shanghai, China). After coating, the plates were incubated in a biochemical incubator (Shanghai Boxun Industry & Commerce Co., Ltd. Medical Equipment, Shanghai, China) at 25 °C for 2 days, followed by inoculation with monoclonal regenerated protoplasts. Clamp collection was monitored after germination, with the absence of clamp connection indicating the mononuclear nature of the protoplasts [16].

### 2.3. PacBio Library Construction and Sequencing

The third-generation sequencing library was constructed by Beijing Beiruihekang Biotechnology Co., Ltd. (Beijing, China). Genomic DNA was fragmented by ultrasound (Shenzhen Kobit ultrasonic equipment Co., Ltd., Shenzhen, China) into 20 kb segments, and the whole genome was sequenced by Pacbio Sequel II (Beijing Beiruihekang Biotechnology Co., Ltd., Beijing, China) with a predicted sequencing depth of 100×.

### 2.4. Hi-C Library Construction and Sequencing

The Hi-C sequencing library was constructed by ANOROAD Gene Technology Co., Ltd. (Beijing, China). [17].

### 2.5. Illumina Library Construction and Sequencing

The second-generation sequencing library was constructed by Beijing Berry Genomics Co., Ltd. (Beijing China) as described. DNA was fragmented by ultrasound into 300 bp segments, followed by double-ended sequencing on an Illumina Hiseq (Beijing Berry Genomics Co., Ltd., Beijing, China) high-throughput sequencing platform. The predicted sequencing depth was 50×, which was used for correction after splicing.

### 2.6. Genome Assembly and Annotation

#### 2.6.1. De Novo Genome Assembly

Clean data sequenced in three generations were assembled de novo using HiFiasm software 0.19.3 [18]. Hi-C data were assembled, with HiCPlotter software2.0 used to correct the sequence, direction, and assembly errors of overlapping groups in the interaction map. Chromosomes were constructed according to interaction hotspots, and telomere sequences were analyzed for chromosome verification. The integrity of genome assembly was assessed by BUSCO (http://busco.ezlab.org (accessed on 1 March 2024), v3.0.2) [19]. The k-mer distribution curve of the genome was determined using mercury v1.3 [20].

#### 2.6.2. Repeat Sequence Annotation

The *H. coralloides* repeat sequence library was built from scratch using Repeat Modeler [21]. Long-terminal repeating retrotransposons were identified using LTRharvest 1.0 [22]. The results were merged using the Repeat Modeler software 1.1.1, and redundancies were removed based on ≥80% consistency using USERACH 2.0 [23]. Based on the RepBase library (http://www.girinst.org/repbase (accessed on 15 March 2024)) and the prediction database, made from scratch, the genomic repeat sequence of *H. coralloides* was predicted using Repeat Masker software 1.08.

#### 2.6.3. Prediction of Non-Coding RNA

Non-coding RNA mainly included tRNA and rRNA. tRNA annotation was analyzed using tRNAscan-SE 2.0 [24]. To annotate rRNA, the rRNA sequence of *H. coralloides* was used as the query sequence, followed by comparison using blastn 1.0.

#### 2.6.4. Annotation of Genome Structure

Gene structure was annotated using three independent methods [25]: de novo prediction, homologous protein-based prediction, and homologous transcript-based prediction.

#### 2.6.5. Annotation of Gene Function

The protein-coding sequences of the genome were searched and matched using the Swiss Protein, Gene Ontology (GO), eukaryotic orthologous group (KOG), Clusters of Orthologous Groups (COGs), Kyoto Encyclopedia of Genes and Genomes (KEGG), and Carbohydrate-Active EnZymes (CAZymes) databases.

### 2.7. Mitochondrial Genome Assembly and Annotation

Mitochondria-related contigs were identified using the BLAST+ program 2.14.0. The circular mitochondrial genome sequence was obtained by cutting out the sequences consistent with the 3′ and 5′ ends and artificially connecting them. Mitochondrial genes were predicted and annotated using the online tool MITOS (http://mitos.bioinf.uni-leipzig.de/index.py (accessed on 20 March 2024)).

### 2.8. Collinearity Analysis

Assembled genomes were compared linearly with other genomes using shinyCircos software v2.0 (https://venyao.xyz/shinyCircos/ (accessed on 2 April 2024)) [26].

### 2.9. Determination of Erinacines

The yield of erinacines was determined by ultraviolet spectrophotometry (Shanghai Yuan analysis Instrument Co., Ltd., Shanghai, China). The standard reference, baicalin, was dissolved in ethanol and diluted to a certain extent, and its ultraviolet absorption spectrum determined by full-UV scanning. Because baicalin showed maximum absorption at 210 nm, its concentration was measured at 210 nm. The concentrations of erinacines were determined relative to a standard working curve for baicalin (Figure S1).

### 2.10. Extraction of Total RNA and Determination of Quality

Total RNA was extracted from samples and its concentration was determined by Omega Bio-tek, Inc. RNA samples were stored at a −80 °C. RNA samples that met the requirements were sent to Wuhan Frasergen Co., Ltd. (Wuhan, China). for quality inspection and library construction. Sequences of clean reads were obtained after quality control and removal of impurities [27]. The amount of clean data for each sample was about 6 G.

### 2.11. Analysis of Differentially Expressed Genes

Using the assembled whole genome of *H. coralloides* as the reference sequence, the levels of expression of each gene were calculated using StringTie v2.1.5, with the results expressed as fragments per kilobase of transcript per million fragments mapped (FPKM) [28]. During the screening process, genes satisfying the criteria *p* < 0.05 and |log2(FC)| > 1 were considered significantly differentially expressed.

### 2.12. Statistical Analysis of Data

Gene function was annotated using Omics Share software 2.0. (https://www.omicshare.com/tools/ (accessed on12 April 2024)), and KEGG and GO enrichment diagrams were drawn using BMK Cloud software 4.8.6.(https://international.biocloud.net/zh/software/tools/list (accessed on 22 April 2024)). Data were analyzed statistically and plotted using GraphPad prism 6.01 and Origin software8.0.

## 3. Results

### 3.1. Culture Conditions

*H. coralloides* strain ‘77’ (DK) was found to have dikaryotic hyphal morphology (Figure 1A). The morphology of DK-derived protoplasts under a 40× microscope was also determined (Figure S2A). Protoplast mononuclearization yielded two strains of mononuclear mycelia with different karyotypes, named ‘77-1’ (MK-1; Figure 1B) and ‘77-2’ (MK-2; Figure 1C), with MK-2 having a faster growth rate than MK-1 under the same culture conditions. The ITS-PCR products of MK-1 and MK-2 DNA had obvious bands at 500–700 bp (Figure S2B). Sequencing of the ITS-PCR products showed that they were 99% similar to the known *H. coralloides* (tvtc0002) ITS sequence, further indicating that both MK-1 and MK-2 were *H. coralloides*.

Evaluation of mycelial morphology under a 100× microscope showed that DK had many clamp connections (Figure 1D), whereas mycelial morphology under a 10× microscope showed that neither MK-1 (Figure 1E) nor MK-2 (Figure 1F) had any clamp connections, suggesting that both MK-1 and MK-2 were monokaryotic strains. Further analysis of DK showed the primordium (FB-A; Figure 1G) and fruiting body (FB; Figure 1H), with yellow arrows pointing to the stipe (FB-B) and spines (FB-C). Because haploids were not only convenient for genome sequencing, analysis, and annotation, but could enable more accurate understanding of the relationships between genes and phenotypes [29,30], genomic DNA was extracted from MK-2 for subsequent sequencing. K-mer distribution curves plotted using Illumina sequencing data (Figure 1I) corresponded to the Poisson distribution and predicted that the MK-2 genome was about 44.8 Mb in size. The curve had a smooth main peak, indicating that the MK-2 genome was highly homozygous, with a small peak to the right of the main peak. The latter was characteristic of a repetitive sequence, indicating that the *H. coralloides* genome had many repetitive sequences, making it difficult to assemble the genome.

### 3.2. High-Quality Genome Assembly

De novo assembly of high quality HiFi reads yielded a total of 67 contig sequences, of average length 681 kb and an N50 of about 3.6 Mb (Table S1). The *H. coralloides* genome was constructed by merging the overlapping sequences. Assembly yielded a genome of about 43.7 Mb in size, consisting of 13 chromosomes and with an N50 of about 3.6 Mb (Table S1).

**Figure 2:**
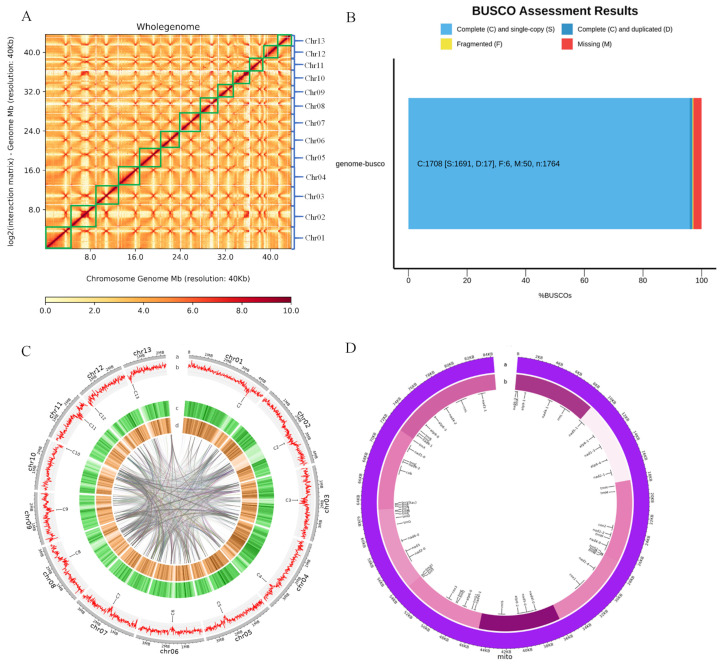
Wholegenome assembly of *H. coralloides*. (**A**) Hi-C interaction map of the whole genome of *H. coralloides,* the green box shows the length of each chromosome. (**B**) Results of BUSCO evaluation. (**C**) Characterization of the *H. coralloides* genome, (a) chromosomes; (b) line chart of GC content; (c) heat map of gene density; (d) heat map of exon content distribution. Internal curve: homologous genes with >98% agreement within the genome were displayed in a wired form. C1-C13: The centromeres of chromosomes 1–13, respectively. (**D**) Characterization of *H. coralloides* mitochondria, (a) mitochondria; (b) distribution map of GC content.

To further verify the level of assembly of the *H. coralloides* genome, Hi-C data were subjected to 3D DNA mounting of the previous assembly results, with and the results of mounting were visualized using HiCPlotter software 2.0. Evaluation of the entire Hi-C genome interaction map, at a resolution of 40 Kb, and with colors from red to white indicating that the interaction relationship ranged from strong to weak, respectively, showed that each chromosome was relatively smooth (Figure 2A). Moreover, the intra-chromosome interactions weakened with distance, indicating that the level of assembly was good.

The integrity and accuracy of the *H. coralloides* genome was evaluated using BUSCO software3.0.2 and the basidiomycetes single copy orthologous gene database (basidiomycota_odb10). The integrity of the assembled *H. coralloides* genome was 96.3%, with only 3.3% missing (Table S2).

The *H. coralloides* genome was 43.7 Mb in size, with 13 chromosomes and 13 centromeres (Table 1 and Figure 2C). About 11,690 genes were predicted, of average length 1566.6 bp. The average number of exons per gene was 7.1, and the average CDS length was 255.94 bp. Although the *H. coralloides* genome was smaller in size and contained fewer chromosomes than plant genomes, genes were more densely distributed in the *H. coralloides* genome than in plant genomes.

Analysis of the mitochondrial genome of *H. coralloides* showed 30 conserved protein-encoding genes, 25 tRNA genes, and 3 small and large subunit rRNA genes (Figure 2D).

Telomeres are specific DNA repeat sequences found at the end of linear chromosomes in eukaryotic cells, usually composed of CCCTAA/TTAGGG (non-transcriptional sequence) [31,32]. Examination of telomere structures at both ends of the chromosomes showed that telomere sequences were reversed and complementary at both ends of Chr01 to Chr13 (Table S3). Moreover, these telomere sequences were found to be CCCAA/TTGGG, slightly different from telomere sequences of most other species. In general, the quality of gene assembly was good, and the complete genome was able to not only provide accurate functional annotation of genes, but enable the evaluation of the evolution and function of complex genomic regions [33].

### 3.3. Prediction of Genome Components

Our analysis predicted a total of 14159 repeats, accounting for 16.52% of the entire genome (Table S4). The lengths of LTR elements in interspersed repeats’ lengths accounted for 7.20% of the genome, whereas the lengths of LINEs and LINE2 accounted for 4.01% and 1.66%, respectively, of the genome. In non-interspersed repeats, simple repeats accounted for 0.72% of the genome, with areas of low complexity and unclassified repetitive sequences accounting for 0.11% and 6.1%, respectively, of the genome.

tRNAscan-SE software2.0 was used to predict the genome sequence of *H. coralloides*. As shown in Table S5, 203 tRNA genes were obtained, with a total length of about 19 kb and corresponding to 20 amino acids. In addition, 45 rRNAs were predicted.

### 3.4. Prediction of Genome Function

**Figure 3:**
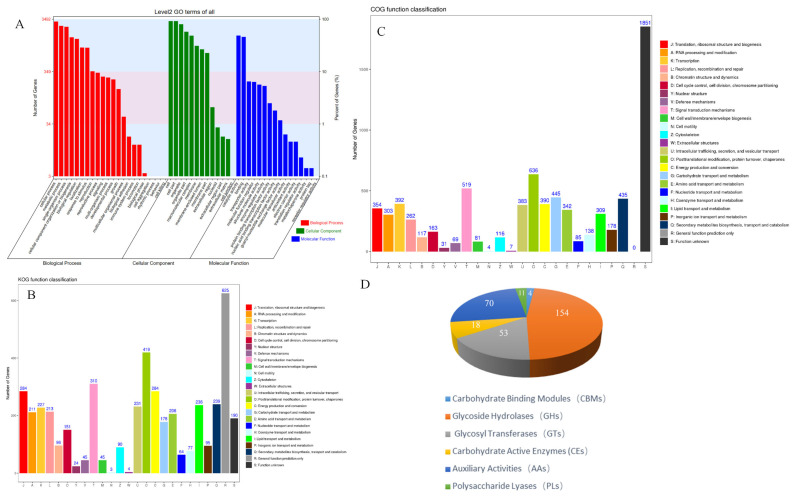
Annotated gene function of *H. coralloides*. (**A**) GO classification. (**B**) KOG classification. (**C**) COG classification. (**D**) CAZymes classification.

The *H. coralloides* genome was functionally annotated using the GO, KEGG, KOG, COG, Swissprot and other databases (Table 2 and Figure 3). A total of 8560 genes were annotated, with 3492 genes annotated by GO, with most of these annotated genes being associated with biological processes. KEGG annotated 4243 genes, belonging to 125 metabolic pathways. KOG annotated 3980 genes, with the largest group (625 genes) predicted to be associated with general function. COG annotated 7090 genes, with the largest group of 1851 being type S genes (function unknown). Pfam, Swissprot, and CAZy annotated 7176, 4949, and 310 genes, respectively, with GHs having the largest number and CBMs having the smallest number of carbohydrate enzymes. These findings indicated that *H. coralloides* could make extensive use of polysaccharides, which was also related to its lifestyle. In addition, 3130 genes could not be annotated.

Six metabolic pathways related to the synthesis of terpenoids by *H. coralloides* were annotated (Table 3). These pathways included those responsible for the biosynthesis of the terpenoid backbone (22), ubiquinone and other terpenoid-quinone complexes (13), diterpenoids (9), sesquiterpenoids and triterpenoids (5), steroids (23), and various types of N-glycan (31). The genes related to terpenoid synthesis accounted for 0.88% of all annotated genes. Many other genes have been assigned to other metabolic pathways, including secondary metabolic pathways, carbon metabolism, and amino acid biosynthesis.

### 3.5. Comparison of the MK-2 Genome with Other Genomes

Comparison of the *H. coralloides* (MK-2) genome analyzed in the present study and a previously identified *H. coralloides* (ASM367540v1) genome showed that the contig N50 in the present study was longer, indicating a longer sequencing reading (Table 4). In addition, the MK-2 genome had a higher GC content and a larger number of predicted genes. Moreover, assembly of the MK-2 genome reached the scale of chromosomes.

**Figure 4:**
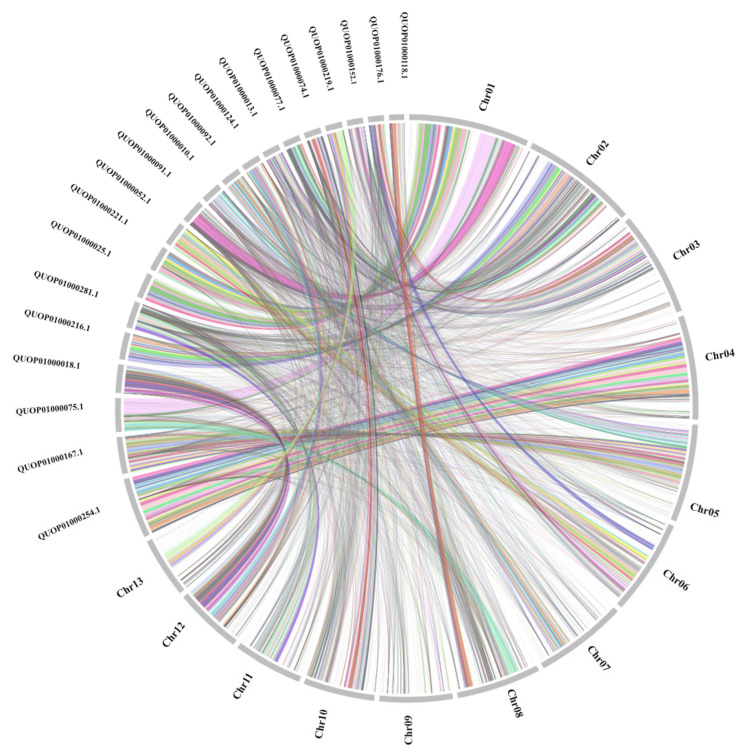
Analysis of the collinearity of the *H. coralloides* (MK-2) and *H. coralloides* (ASM367540v1) genomes. The 20 largest contigs of ASM367540v1 were compared with DK chromosomes.

Genome collinearity analysis comparing the MK-2 genome and the 20 largest contigs of the *H. coralloides* (ASM367540v1) genome (Figure 4) showed a high degree of collinearity. These findings indicate that genome assembly was more accurate and of higher quality in the present study than in previous analyses.

### 3.6. Prediction of H. Coralloides Gene Clusters Related to the Biosynthesis of Terpenoids

Terpenoids are the largest type of natural product, with more than 50,000 known structures. These compounds are derived from isoprene pyrophosphate (IPP) and dimethyl allyl pyrophosphate (DMPP) [34].

In fungi, a set of tightly linked and functionally closely related structural genes is called a gene cluster. These genes were dispersed in a discrete manner around the core genes [35]. The complexity and variability of these clusters made them difficult to fully characterize. In this study, genes and gene clusters associated with terpenoid biosynthesis were predicted by KEGG pathway analysis (https://www.genome.jp/kegg/ (accessed on 31 August 2024)), homologous sequence searching (BLAST) and antiSMASH 3.0 software. Genes related to terpenoid backbone biosynthesis were identified by KEGG pathway annotation. Because GGPPS (geranylgeranyl pyrophosphate synthase1) is usually the core gene in the diterpenoid biosynthesis gene clusters of most fungi and bacteria, BLAST was used to predict the gene sequences located 20 kb upstream and downstream of GGPPS sequences, with some of the predicted genes being diterpenoid gene clusters. Sesquiterpene synthases were identified based on homologous sequence searching of 6 STS (Cop1-6) in the model Basidiomycetes Coprinus cinereus, followed by phylogenetic analysis [36]. Finally, gene clusters associated with the synthesis of secondary metabolites were predicted using antiSMASH 3.0 software.

### 3.7. Terpenoid Backbone Biosynthesis Pathway

Differences in gene expression were assessed using the transcriptomes of *H. coralloides* strains DK ‘77’, MK-1, and MK-2, and the primordium (FB-A), stipe (FB-B), and spines (FB-C). KEGG enrichment of differentially expressed genes (DEGs) in MK-1 and MK-2 identified 275 DEGs, including 58 up-regulated and 217 down-regulated DEGs (Figure S3A,B). A comparison of DK with FB-A identified 494 DEGs, including 230 up-regulated and 264 down-regulated DEGs (Figure S3C,D), whereas a comparison of DK and FB-B identified 471 DEGs, including 196 up-regulated and 275 down-regulated DEGs (Figure S3E,F). A comparison of DK and FB-C identified 534 DEGs, including 203 up-regulated and 331 down-regulated DEGs (Figure S3G,H). Most DEGs were enriched in metabolic pathways, indicating that changes in terpenoids may be related to these metabolic pathways (Figure S4).

**Figure 5:**
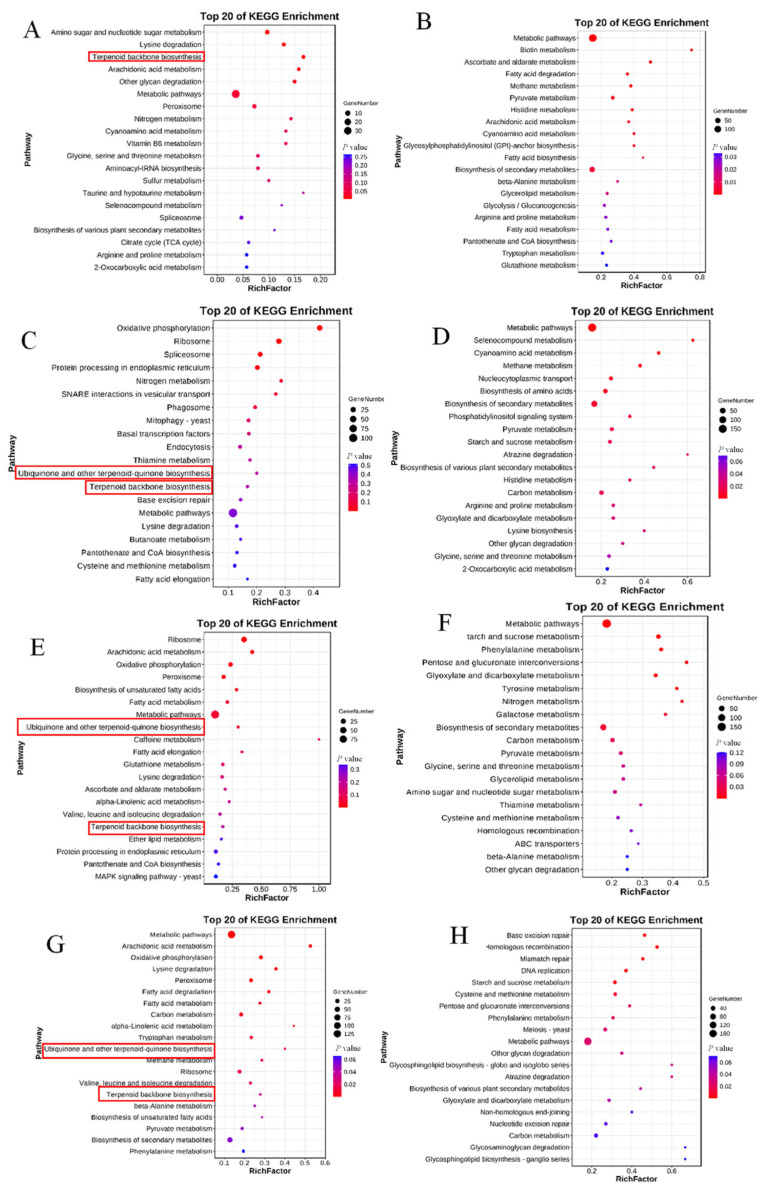
KEGG enrichment analysis of differentially expressed genes (DEGs) in *H. coralloides*. (**A**,**B**) DEGs up-regulated (**left**) and down-regulated (**right**) in comparisons of MK-1 and MK-2. (**C**,**D**) DEGs up-regulated (**left**) and down-regulated (**right**) in comparisons of DK and FB-A. (**E**,**F**) DEGs up-regulated (**left**) and down-regulated (**right**) in comparisons of DK and FB-B. (**G**,**H**) DEGs up-regulated (**left**) and down-regulated (**right**) in comparisons of DK and FB-C. The red boxes indicate terpenoid-related synthetic pathways.

The DEGs most closely associated with terpenoids were analyzed by determining the top 20 KEGG pathways in each pairwise comparison (Figure 5). In each comparison, the up-regulated genes (*Her005768*, *Her008335,* and *Her011461*) were associated with the terpenoid backbone biosynthesis pathway (ko00900). *Her005768* was found to encode 4-diphosphocytidyl-2-C-methyl-D-erythritol kinase (iSPE), whereas *Her008335* and *Her011461* were found to encode farnesyl diphosphate synthase (FDPS)/geranylgeranyl pyrophosphate synthase1 (GGPPS) (Table S6); FDPS and GGPPS are key enzymes in the biosynthesis of geranylgeranyl pyrophosphate (GGPP), a common precursor in diterpene synthesis. iSPE, FDPS, and GGPPS have been reported to be important rate-limiting enzymes in the biosynthesis of terpenoids [37]. For example, the expression of genes regulating iSPE, FDPS, and GGPPS in Wolfiporia cocos was up-regulated in the mycelial stage, a finding consistent with alterations in triterpenoid production and suggesting that genes regulating iSPE, FDPS, and GGPPS are involved in the biosynthesis of triterpenes in the mycelial stage [38]. Similarly, FDPS, GGPPS, and iSPE likely played a positive role in terpenoid synthesis in the present study. Compared with MK-2, the levels of expression of genes encoding FDPS, GGPPS, and iSPE were 2.00-, 2.41-, and 3.81-fold higher, respectively, in MK-1. Similarly, the levels of expression of the genes encoding FDPS, GGPPS, and iSPE were 3.56-, 4.4-, and 2.77-fold higher, respectively, in DK than in FB-A; 2.83-, 3.32-, and 4.92-fold higher, respectively, in DK than in FB-B; and 4.03-, 3.92-, and 5.86-fold higher, respectively, in DK than in FB-C (Table 5).

Comparisons of DK with FB-A, FB-B, and FB-C comparison groups identified two, three, and three up-regulated genes, respectively, that were significantly enriched in ubiquinone and terpenoid–quinone biosynthesis (ko00130) (Figure 5, Table S6 and Table 5). The two genes in comparisons of DK with FB-A (*Her010901* and *Her001132*) were found to encode 4-hydroxybenzoate polyprenyltransferase (ubiA)/4-hydroxybenzoate polyprenyltransferase (COQ2) and 3-demethoxyubiquinol 3-hydroxylase (ubiF)/3-demethoxyubiquinol 3-hydroxylase (COQ7); the three genes in comparisons of DK with FB-B (*Her010901*; *Her009959*; and *Her001132*) were found to encode ubiA/COQ2 and ubiF/COQ7, and the three genes in comparisons of DK with FB-C (*Her006816*; *Her010901*; and *Her009959*) were found to encode tyrosine aminotransferase (TAT)/aromatic amino acid aminotransferase I/2-aminoadipate transaminase (ARO8), and ubiF/COQ7, ubiA/COQ2 and TAT/ARO8, enzymes involved in the synthesis of ubiquinone and vitamin E. COQ2, a polymeric membrane protein of the UbiA terpenoid cyclase superfamily, was found to be essential for the biosynthesis of ubiquinone (COQ) in Saccharomyces cerevisiae, as this enzyme catalyzed the pentenylation of p-hydroxybenzoate and all-trans polypentenyls; making terpenoid cyclase (COQ2) was crucial for the biosynthesis of ubiquinone (COQ) [39]. Genes encoding COQ2 and TAT were found to participate in the regulation of terpenoids and to be core genes in terpenoid biosynthesis pathways [40], suggesting that the COQ2 and TAT genes were related to the synthesis of terpenoids.

Comparisons of MK-1 and MK-2 showed that FDPS, GGPPS, and iSPE genes were significantly up-regulated in MK-1. Comparisons of DK with FB-A, FB-B, and FB-C showed that the FDPS, GGPPS, iSPE, COQ2, COQ7, and TAT genes were usually up-regulated in DK. These findings indicated that terpenoids may be more abundant in MK-1 than in MK-2 and may be more abundant in DK than in FB-A, FB-B, and FB-C.

### 3.8. Diterpenoid Biosynthesis Pathway

*H. coralloides* produces erinacines, a class of cyathane diterpenoid compounds. The direct precursor of diterpenoid biosynthesis is GGPP, formed by the condensation of the common precursors IPP and DMAPP and giving rise to larger structural units, including GPP(C10), FPP(C15) and GGPP(C20). Diterpenoid synthases catalyze the synthesis of diterpenoid compounds from GGPP via the mevalonate (MEP) pathway. Different types of diterpenoid synthase are responsible for the synthesis of a variety of diterpenoid compounds. Diterpenoid synthase (ditps), diterpenoid cyclase, and cytochrome p450 are the key diterpenoid synthase enzymes in fungi.

To identify genes involved in diterpenoid biosynthesis in *H. coralloides*, the GGPPS sequence of the basidiomycetes species *Clitopilus passeckerianus* was used to search for homologous amino acid sequences in the *H. coralloides* genome [41,42]. After identifying a gene (*Her011461*) encoding GGPPS, the sequences located 20 kb upstream and downstream of *Her011461* were subjected to BLAST analysis. Ten genes, forming diterpene biosynthesis gene clusters, were identified (Table 6).

The predicted gene cluster for diterpenoid biosynthesis was located on chromosome 13 and included one GGPPS, three cytochrome p450, two NAD(P) binding proteins, one UDP glycosyltransferase, one diterpene cyclase, one ABC transporter, and one ubiA pentenyltransferase. Comparison of the MK-1 and MK-2 strains showed that the levels of expression of genes encoding NADP binding protein (*Her011458*), diterpene cyclase (*Her011463*), UbiA prenyltransferase (*Her011463*), NAD binding protein (*Her011465*), and UDP-glycosyltransferase (*Her011467*) were significantly higher in MK-2 than in MK-1, suggesting that diterpenoids may be more abundant in MK-2 than in MK-1. The levels of expression of three cytochrome P450 (*Her011457*, *Her011459,* and *Her011466*) encoding genes did not differ significantly in FB-A, FB-B, FB-C, and DK. In contrast, the levels of expression of genes encoding geranylgeranyl pyrophosphate synthase (*Her011461*), NAD binding protein (*Her011465*) and UDP-glycosyltransferase (*Her011467*) were significantly higher in DK than in FB-A, FB-B, and FB-C, further indicating that diterpenoid content may be higher in DK than in FB-A, FB-B, and FB-C. Similar results were observed in previous studies.

**Figure 6:**
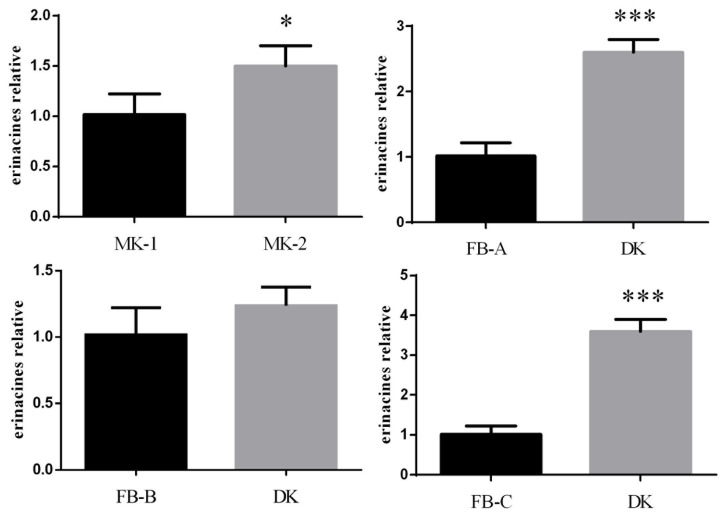
Histogram of the relative content of erinacines (* *p* < 0.05, *** *p* < 0.001 by paired *t*-tests).

Because fungi contain many types of diterpenoids and the extraction methods are difficult, differences in diterpenoid contents among strains were verified by assaying the amounts of erinacine, a representative diterpenoid in *H. coralloides*. The contents of erinacine in DK were 2.62-, 1.23-, and 3.6-fold higher than in FB-A, FB-B, and FB-C, respectively, and 1.5-fold higher in MK-2 than in MK-1 (Figure 6).

### 3.9. Sesquiterpene Biosynthesis Pathway

Sesquiterpenes are important secondary metabolites of basidiomycetes, of various structural types and physiological and ecological functions. Sesquiterpene synthases (STSs) are key enzymes in the formation of the sesquiterpene backbone. STSs of different species vary in structures and catalytic mechanisms, resulting in the structural diversity of sesquiterpenes. Although many studies have described sesquiterpene compounds in basidiomycetes, few studies have described STSs in these organisms.

**Figure 7:**
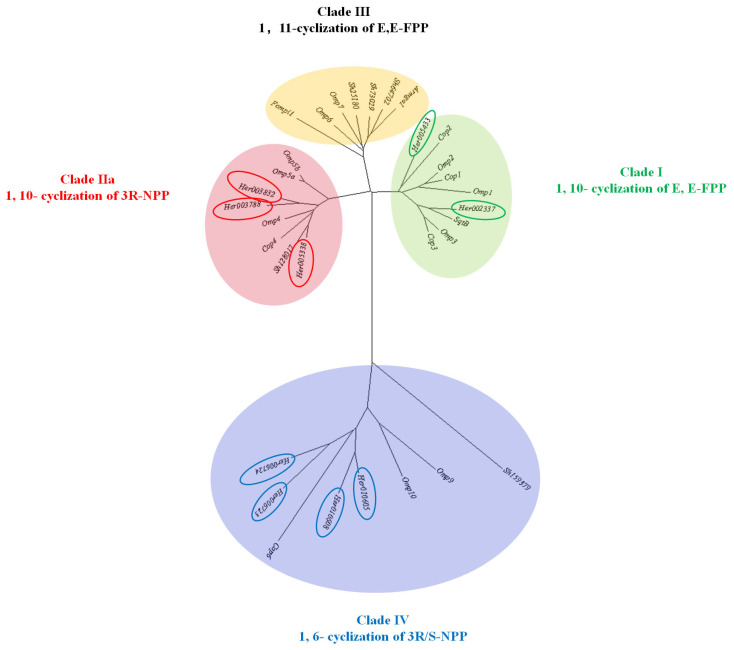
Unrooted neighbor-joining phylogram of sesquiterpene synthases (STSs) of *H. coralloide*, *Coprinopsis cinereus* (Cop), *Omphalotus olearius* (Omp), *Fomitopsis pinicola* (Fompi1), *Stereum hirsutum* (Sh), *Armillaria gallica* (Armga1), and *Boreostereum vibrans* (SqtB). Construction was based on homologous protein sequences. Details of the sequences included in the phylogenetic tree are described in Data S1. The genes circled are the ones predicted in this paper.

To identify genes encoding STSs in *H. coralloides*, a homology search was *H. coralloides* performed using STS genes (Cop1-6) from the model basidiomycetes species *Coprinus cinereus* [43]. This search identified nine *H. coralloides* genes possibly encoding STSs (*Her003832*, *Her1003788*, *Her005338*, *Her002337*, *Her005433*, *Her006723*, *Her006724*, *Her010605*, and *Her010608*), located on five chromosomes (Chr02, Chr04, Chr05, Chr06, and Chr12). Preliminary phylogenetic analysis showed that these genes belonged to four clades (clades I-IV), suggesting that the STSs encoded by genes in each group may produce related sesquiterpenes via conserved cyclization pathways. The nine genes encoding STSs in *H. coralloides* belonged to three clades (clades I, II, and IV), representing three functional groups. Two of these genes, in clade I, encoded alpha-muurolene synthase (*Her002337*, *Her005433*), three genes in clade II encoded linoleate 10R-lipoxygenasexygenase (*Her1003788*, *Her003832,* and *Her005338*), and four genes in clade IV encoded trichodiene synthase (*Her006723*, *Her006724*, *Her010650*, and *Her010608*) (Figure 7, Data S1) [44,45]. Most of the nine genes encoding STSs were down-regulated in the four comparison groups. The four genes encoding trichodiene synthase (*Her006723*, *Her006724*, *Her010650*, and *Her010608*) were significantly down-regulated in all groups, with the levels of expression of *Her006724*, *Her010605,* and *Her010608* being 9.25-, 5.82-, and 3.48-fold lower, respectively, in DK than in FB-B, and 14.22-, 4.23-, and 2.60-fold lower, respectively, in DK than in FB-C.

**Figure 8:**
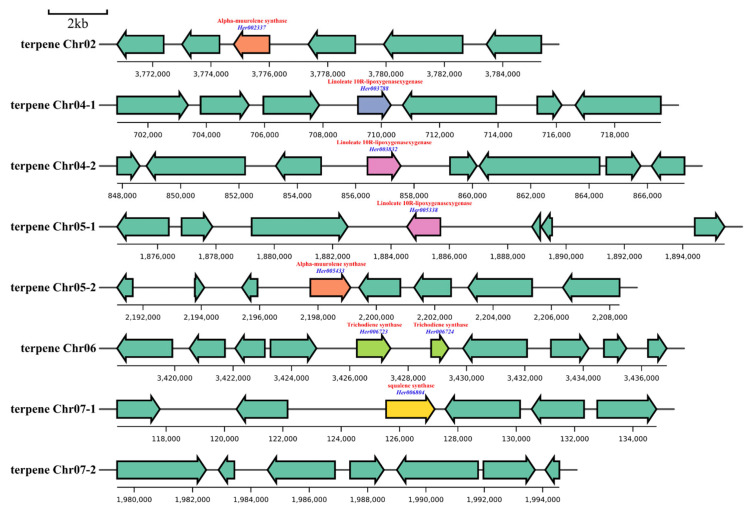
Identification of terpenoid gene clusters by antiSMASH software. The map includes functional annotations (red) and gene names (blue). Details for the annotation of each gene cluster are shown in Table S7.

antiSMASH software identified eight gene clusters involved in terpenoid biosynthesis. These clusters were located on five chromosomes (Chr02, Chr04, Chr05, Chr06, and Chr07), with each cluster spanning 20 kb of the *H. coralloide* genome (Figure 8 and Table S7). The Chr02 gene cluster included a gene (*Her002337*) encoding alpha-muurolene synthase; the Chr04-1 cluster included a gene (*Her003788*) encoding linoleate 10R-lipoxygenasexygenase; and the Chr04-2 gene cluster included a gene (*Her003832*) encoding linoleate 10R-lipoxygenasexygenase. In addition, the Chr05-1 gene cluster contained a gene (*Her005338*) encoding linoleate 10R-lipoxygenasexygenase; the Chr05-2 cluster contained a gene (*Her005433*) encoding alpha-muurolene synthase; the Chr06 cluster contained genes (*Her006723*, *Her006724*) encoding trichodiene synthase; and the Chr07-1 cluster included a gene (*Her006804*) encoding squalene synthase. Thus, most of the predicted genes were involved in sesquiterpene synthesis, and there were no genes involved in the terpenoid backbone biosynthesis pathway and diterpenoid biosynthesis pathway. Results obtained using antiSMASH software differed from those of homology searching for terpenoid synthesis gene clusters. Although antiSMASH could be used to identify specific PKS and NRPS gene clusters in ascomycetes or bacteria, this method was not suitable for identifying terpenoid gene clusters in basidiomycota. Therefore, genes related to the terpenoid backbone, diterpenoid, and sesquiterpene biosynthesis pathways were identified by homology searching, as well as by the use of antiSMASH software.

### 3.10. Validation of RNA-Seq Results Using qRT-PCR

**Figure 9:**
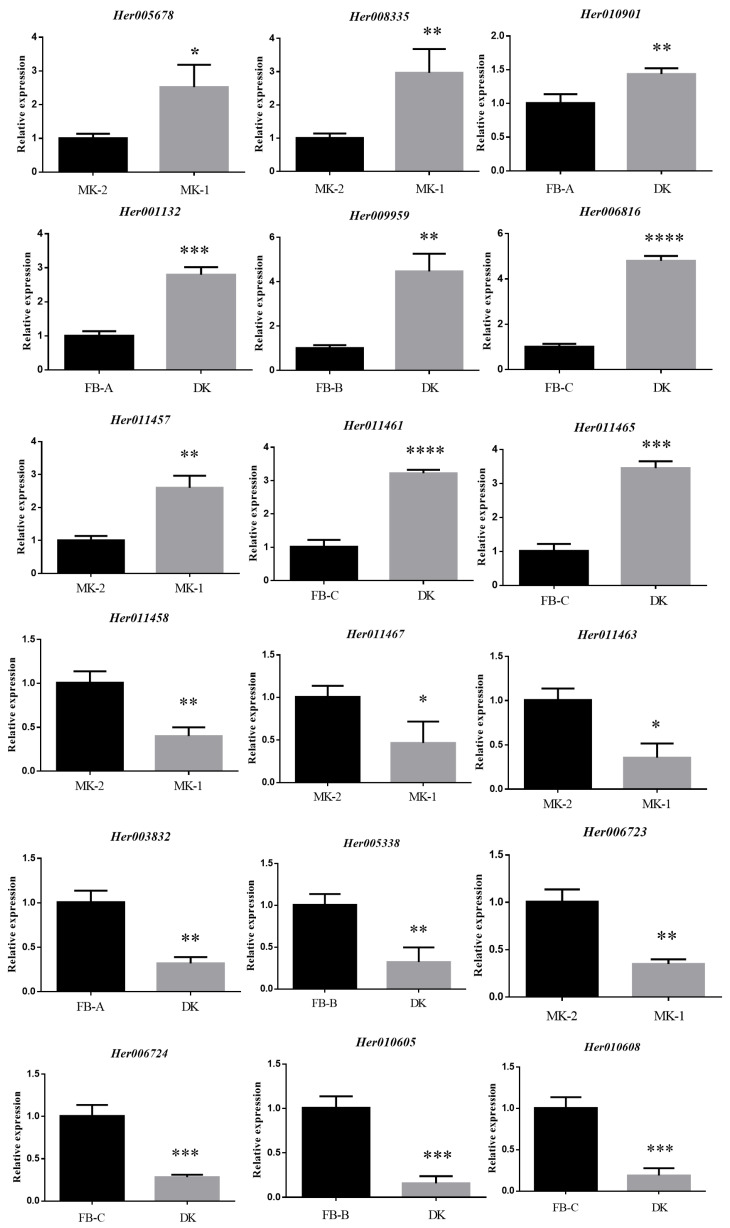
Validation of genes changes by qRT-qPCR (* *p* < 0.05, ** *p* < 0.01, *** *p* < 0.001, **** *p* < 0.0001 by paired *t*-tests).

The accuracy of RNA-Seq results was verified by real-time quantitative polymerase chain reaction (qRT-PCR). DEGs selected in the terpenoid backbone synthesis pathway (*Her005678*, *Her008335*, *Her010901*, *Her001132*, *Her006816*, and *Her009959*) were those up-regulated in MK-1 and DK. DEGs selected in the diterpenoid biosynthesis pathway included those both up-regulated (*Her011457*) and down-regulated (*Her011458*, *Her011463,* and *Her011467*) in MK-1 and genes up-regulated (*Her011461* and *Her011465*) in DK. DEGs selected in the sesquiterpene biosynthesis pathway included those down-regulated (*Her003832*, *Her005338*, *Her006723*, *Her006724*, *Her010605*, and *Her010608*) in both MK-1 and DK. The qRT-PCR results of these genes were consistent with the results of transcriptome analysis (Figure 9), indicating that the RNA-Seq data were accurate and reliable.

## 4. Discussion

This study describes the sequencing, assembly, and annotation of the *H. coralloides* genome, finding that this genome is 43.7 Mb in size, with 13 chromosomes and an N50 of 3.60 Mb. The effect of chromosome assembly evaluated by BUSCOs was 96.9%. Compared with the previously described ASM367540v1 genome, the *H. coralloides* genome had higher degrees of continuity and integrity.

Many genes associated with terpenoid biosynthesis were identified by homologous comparison and KEGG enrichment analysis. Geranylgeranyl pyrophosphate synthase (GGPPS) and farnesyl diphosphate synthase (FDPS), encoded by the *Her011461* and *Her008335* up-regulated genes in the terpenoid backbone synthesis pathway (ko00900), respectively, are key enzymes in the biosynthesis of GGPP, a common precursor of diterpenes. In addition, the expression level of these genes, *Her011461* (Geranylgeranyl pyrophosphate synthase), *Her011465* (NAD binding protein), and *Her011467* (UDP-glycosyltransferase) in DK was significantly up-regulated compared with FB-A, FB-B, and FB-C in the diterpenoid biosynthesis pathway. This indicated that the content of diterpenoids in DK might be higher than FB-A, FB-B, and FB-C. A total of nine presumed STS genes were identified in the sesquiterpene synthesis pathway (*Her003832*, *Her1003788*, *Her005338*, *Her002337*, *Her005433*, *Her006723*, *Her006724*, *Her010605*, and *Her010608*). Most of the nine STS genes were down-regulated in the comparison groups. In addition, genes involved in sesquiterpene biosynthesis were found and dispersed in the *H. coralloide* genome. In general, these genes show different levels of expression in different tissues.

Transcriptome analysis showed that the genes responsible for terpenoid backbone and diterpenoid biosynthesis were highly expressed in DK, whereas all nine genes responsible for sesquiterpenoid biosynthesis were under-expressed in DK (Table 7), suggesting that gene expression may be related to tissue-specific biosynthesis of active compounds. These results also indicated that, under our culture conditions, *H. coralloides* may produce very small amounts of sesquiterpenoids.

The eight gene clusters involved in terpenoid biosynthesis were identified using antiSMASH software, with most of the predicted genes in these clusters being involved in sesquiterpene biosynthesis. In contrast, these clusters did not include genes involved in terpenoid backbone or diterpene biosynthesis. These findings indicated that the two methods of predicting terpenoid synthesis gene clusters yielded different results, suggesting that both the homology search and antiSMASH software methods were required to identify genes associated with the terpenoid backbone, diterpenoid, and sesquiterpenoid biosynthesis.

In summary, the results of this study provide additional genomic information on *H. coralloides*, including the identification of key genes involved in terpenoid biosynthesis. These findings not only increase understanding of *H. coralloide* biology, but suggest the possible commercial application of terpenoid metabolites from *H. coralloide*.

## Figures and Tables

**Table 1:** Statistics of *H. coralloides* genome assembly results.

Assembly Feature	*H. coralloides*
Number of Chromosome	13
Assembly size (Mb)	43.7
Assembly of genome (%)	96.9%
Number of predicted gene models	11,690
Average coding sequence length (bp)	255.94
Average gene length (bp)	1, 566.6
Average exons per gene	7.1

**Table 2:** Summary of *H. coralloides* genome annotation.

Database	Number	Percent (%)
GO_Annotation	3492	29.9
KEGG_Annotation	4243	36.3
KOG_Annotation	3980	34.0
COG_Annotation	7090	60.7
Pfam_Annotation	7176	61.4
CAZy	310	2.7
Swissprot_Annotation	4949	42.3
All_Annotated	8560	73.2
Unannotated	3130	26.8
Total	11,690	-

**Table 3:** Metabolic pathways and genes related to the synthesis of terpenoids in *H. coralloides*.

Terpenoid-Related Pathways	Genes Numbers	Percentage (%)
Terpenoid backbone biosynthesis	22	0.19%
Ubiquinone and other terpenoid–quinone biosynthesis	13	0.11%
Sesquiterpenoid and triterpenoid biosynthesis	5	0.04%
Steroid biosynthesis	23	0.2%
N-glycan biosynthesis	31	0.27%
Other glycan degradation	19	0.16%
Various types of N-glycan biosynthesis	22	0.19%

**Table 4:** Comparison of genome information of the *H. coralloides* (MK-2) and *H. coralloides* (ASM367540v1) genomes.

Assembly Feature	*H. coralloides* (MK-2)_PacBio	*H. coralloides* (ASM367540v1)_PacBio
Assembly size (Mb)	43.7	55.8
Number of contigs	63	306
Contig N50 (bp)	3,598,748	441,100
Number of scaffolds	13	-
Scaffold N50 (bp)	3,598,748	-
GC percent (%)	54	53.5
Number of predicted gene models	11,690	13,175
Assembly level	Chromosome	Contig

**Table 5:** Genes and enzymes of the terpenoid backbone biosynthesis pathway.

Gene ID	Function	Chromosome	Log2 Ratio MK-1/MK-2	Log2 Ratio DK/FB-A	Log2 Ratio DK/FB-B	Log2 Ratio DK/FB-C
*Her005768*	4-diphosphocytidyl-2-C-methyl-D-erythritol kinase	Chr05	1.00	1.83	1.50	2.01
*Her008335*	FDPS/GGPPS	Chr08	1.27	2.14	1.73	1.97
*Her011461*	FDPS/GGPPS	Chr13	1.93	1.47	2.30	2.55
*Her010901*	ubiA/COQ2	Chr12	−0.02	2.92	2.59	2.71
*Her009959*	ubiA/COQ2	Chr11	0.51	0.01	2.82	1.31
*Her001132*	ubiF/COQ7	Chr01	0.14	1.46	1.21	1.53
*Her006816*	TAT/ARO8	Chr07	−0.26	0.12	0.93	1.27

MK-1 and MK-2: mononuclear mycelium; DK: dikaryotic mycelium; FB-A: primitive stage; FB-B: fruiting body stalk; FB-C: fruiting body spines. Orange indicates a significantly up-regulated gene; gray indicates no significant difference in expression.

**Table 6:** Genes and enzymes of diterpenoid biosynthesis pathway.

Gene ID	Function	Chromosome	Log2 Ratio MK-1/MK-2	Log2 Ratio DK/FB-A	Log2 Ratio DK/FB-B	Log2 Ratio DK/FB-C
*Her011457*	cytochrome p450	Chr13	1.04	0.52	0.40	0.29
*Her 011458*	NADP binding protein	Chr13	−1.76	0.22	0.76	1.16
*Her011459*	cytochrome p450	Chr13	−0.61	−0.55	0.41	0.25
*Her011460*	ABC-transporter	Chr13	−0.30	−0.98	032	0.74
*Her011461*	Geranylgeranyl pyrophosphate synthase	Chr13	1.93	1.47	2.30	2.55
*Her011463*	diterpene cyclase	Chr13	−2.30	−0.22	−0.62	0.24
*Her011463*	UbiA prenyltransferase	Chr13	−2.30	−0.22	−0.62	0.24
*Her011465*	NAD binding protein	Chr13	−1.08	1.55	1.67	3.77
*Her011466*	cytochrome p450	Chr13	−0.33	−0.623	−0.60	0.06
*Her011467*	UDP-glycosyltransferase	Chr13	−1.24	0.034	1.04	2.38

MK-1 and MK-2: mononuclear mycelium; DK: dikaryotic mycelium; FB-A: primitive stage; FB-B: fruiting body stalk; FB-C: fruiting body spines. Orange indicates a significantly up-regulated gene; green indicates a significantly down-regulated gene; gray indicates no significant difference in expression.

**Table 7:** Genes and enzymes of sesquiterpene biosynthesis pathway.

Gene ID	Function	Chromosome	Log2 Ratio MK-1/MK-2	Log2 Ratio DK/FB-A	Log2 Ratio DK/FB-B	Log2 Ratio DK/FB-C
*Her003788*	Linoleate 10R-lipoxygenasexygenase	Chr04	−0.66	0.57	−0.26	−0.10
*Her003832*	Linoleate 10R-lipoxygenasexygenase	Chr04	−1.55	−1.88	−0.07	−1.00
*Her005338*	Linoleate 10R-lipoxygenasexygenase	Chr05	−0.91	−0.04	−1.54	−0.99
*Her002337*	Alpha-muurolene synthase	Chr02	−0.79	−0.42	−0.45	0.45
*Her005433*	Alpha-muurolene synthase	Chr05	−0.47	−0.14	−1.37	−1.24
*Her006723*	Trichodiene synthase	Chr06	−2.60	0.44	0.24	−0.07
*Her006724*	Trichodiene synthase	Chr06	0.87	−1.69	−3.21	−3.83
*Her010605*	Trichodiene synthase	Chr12	1.51	−1.38	−2.54	−2.08
*Her010608*	Trichodiene synthase	Chr12	−2.63	−0.88	−1.8	−1.38

MK-1 and MK-2: mononuclear mycelium; DK: dikaryotic mycelium; FB-A: primitive stage; FB-B: fruiting body stalk; FB-C: fruiting body spines. Orange indicates a significantly up-regulated gene; green indicates a significantly down-regulated gene; gray indicates no significant difference in expression.

## Data Availability

All experimental data in this study will be made available upon reasonable request from readers.

## Supplementary Materials

The following supporting information can be downloaded at: https://www.mdpi.com/article/10.3390/jof10100704/s1: Figure S1: Calibration plot of scutellarin; Figure S2: Protoplast mononuclearization results of DK. (**A**) Morphology of protoplasts DK under 40× microscope, the protoplasts are circled in red. (**B**) Electropherogram of ITS-PCR. Figure S3: KEGG enrichment analysis of DEGs under different groups. (**A**,**B**) Up-regulated DEGs and down-regulated DEGs in MK-1 and MK-2 comparison groups. (**C**,**D**) Up-regulated DEGs and down-regulated DEGs in DK and FB-A comparison groups. (**E**,**F**) Up-regulated DEGs and down-regulated DEGs in DK and FB-B comparison groups. (**G**,**H**) Up-regulated DEGs and down-regulated DEGs in DK and FB-C comparison groups; Figure S4: KEGG pathway annotation of DEGs in different groups. (**A**,**B**) Up-regulated DEGs (**left**) and down-regulated DEGs (**right**) in MK-1 and MK-2 comparison groups. (**C**,**D**) Up-regulated DEGs (**left**) and down-regulated DEGs (**right**) in DK and FB-A comparison groups. (**E**,**F**) Up-regulated DEGs (**left**) and down-regulated DEGs (**right**) in DK and FB-B comparison groups. (**G**,**H**) Up-regulated DEGs (**left**) and down-regulated DEGs (**right**) in DK and FB-C comparison groups. Table S1**:** Count of HiFi reads and de novo assembly contigs; Table S2: *H. coralloides* genome assessed by BUSCO pipeline; Table S3: Statistics of telomeres of the both ends of chromosomes in *H. coralloides*; Table S4: Classification of repetitive sequences in *H. coralloides*; Table S5: Statistics of tRNA in *H. coralloides*; Table S6: Metabolic pathways and gene distribution related to the synthesis of terpenoids in each group; Table S7: Identification of the putative gene clusters for terpene in *H. coralloide* genome; Data S1: The amino acid coding sequence of genes.
