# Supplementary material for: Chromosome-Scale Genome and Transcriptomic Analyses Reveal Differential Regulation of Terpenoid Secondary Metabolites in Hericium coralloides
Source: J Fungi (Basel). 2024 Oct 9;10(10):704. doi: 10.3390/jof10100704 (PMC11508549; doi:10.3390/jof10100704)
Supplement: Supplementary file 1 [file jof-10-00704-s001.zip › 3-Supplementary Materials .pdf]

Supplementary materials:

Chromosome-scale genome and transcriptomic analyses reveal differential regulation of terpenoid secondary metabolites in *Hericium coralloides*

Kexin Meng[1,2], Junyi Lv[1,2], Tuo Zhang[1,2],Yuanyuan Liu[1,2], Peng Zhang[1,2], Yue Zhang[1,2], Banghui Hu[1,2], Qianhui Huang[3*],Baogui Xie[2*], Junsheng Fu[1,2*]

[1] College of Life Sciences, Fujian Agriculture and Forestry University,Fuzhou 350002, China;MKX15736938616@163.com (K.M.); lovesicklqf@Outlook.com (J.L.)； zhangtuo_1001@163.com (T.Z.); lyylyy0815@163.com (Y.L.); 1960390216@qq.com (P.Z.); great_zhangyue@163.com (Y.Z.); 1918210324@qq.com (B.H.); dengyoujin1980@163.com(Y.D.); fujunsheng81@163.com(J.F)

[2] Mycological Research Center, Fujian Agriculture and Forestry University,Fuzhou 350002, China; mrcfafu@163.com (B.X.)

[3] College of Biological Sciences and Engineering, Ningde Normal University, Ningde 352100, China; elberthuang123@163.com (Q.H.)

* Correspondence: fujunsheng81@163.com(J.F); mrcfafu@163.com (B.X.); elberthuang123@163.com (Q.H.)

Table S1. Count of HiFi reads and de novo assembly contigs

Term	*De novo* assembly conting count	Genome assembly results
**Total number**	67	13
**Total bases/ bp**	45 682 370	43 651 881
**Minimum length/ bp**	22 720	2 270 414
**Maximum length/ bp**	4 558 022	4 558 022
**Average length/ bp**	681 826	3 357 837
**N50/ bp**	3 598 748	3 598 748
**N90/ bp**	2 561 033	2 561 033

Table S2. *H. coralloides* genome assessed by BUSCO pipeline

Type of assessment	basidiomycota（1764 BUSCOs）	
	**Number**	**Percent**
**Complete BUSCOs**	1708	96.90%
**Complete and single-copy BUSCOs**	1691	95.90%
**Complete and duplicated BUSCOs**	17	1.00%
**Fragmented BUSCOs**	6	0.30%
**Missing BUSCOs**	50	2.80%

Table S3. Statistics of telomeres of the both ends of chromosomes in *H. coralloides*

SeqID	SeqLength	Start	End	Length	Type
**Chr01**	4558022	20	159	140	CCCAA
**Chr01**	4558022	4557882	4558021	140	TTGGG
**Chr02**	4387448	5	149	145	CCCAA
**Chr02**	4387448	4387276	4387443	168	TTGGG
**Chr03**	3953495	10	144	135	CCCAA
**Chr03**	3953495	3953356	3953495	140	TTGGG
**Chr04**	3905799	6	148	143	CCCAA
**Chr04**	3905799	3905643	3905798	156	TTGGG
**Chr05**	3675261	6	118	113	CCCAA
**Chr05**	367526	3675110	3675260	151	TTGGG
**Chr06**	3598748	6	154	149	CCCAA
**Chr06**	3598748	3598614	3598747	134	TTGGG
**Chr07**	3477545	6	149	144	CCCAA
**Chr07**	3477545	3477437	3477541	105	TTGGG
**Chr08**	3190813	6	158	153	CCCAA
**Chr08**	3190813	3190682	3190809	128	TTGGG
**Chr09**	2756616	5	132	128	CCCAA
**Chr09**	2756616	2756472	2756615	144	TTGGG
**Chr10**	2699080	12	161	150	CCCAA
**Chr10**	2699080	2698923	2699079	157	TTGGG
**Chr11**	2617607	2	141	140	CCCAA
**Chr11**	2617607	2617514	2617603	90	TTGGG
**Chr12**	2561033	6	150	145	CCCAA
**Chr12**	2561033	2560878	2561032	155	TTGGG
**Chr13**	2270414	6	144	139	CCCAA
**Chr13**	2270414	2270305	2270410	106	TTGGG

Table S4. Classification of repetitive sequences in *H. coralloides*

Class	number	Length	Percentage
**Total**	14159	7212072	16.52
**Non-interspersed Repeats**	6866	362813	0.83
**Simple-repeat**	6032	316302	0.72
**Low-complexity**	834	46511	0.11
**Small RNA**	0	0	0
**Satellite**	0	0	0
**Total interspersed repeats**	7293	6870773	15.74
**SINEs:**	27	2389	0.01
**LTR elements**	2490	3145094	7.2
**LINEs**	110	767579	1.76
**LINE2**	92	724704	1.66
**DNA elements**	551	293055	0.67
**Unclassified**	4023	2662656	6.1

Table S5. Statistics of tRNA in *H. coralloides*

**Amino acid**	**Type1**	**Type2**	**Type3**	**Type4**
**Ala/15**	AGC/7	GGC/1	CGC/4	TGC/3
**Arg/19**	ACG/8	-	CCG/2<br>CCT/2	TCG/4<br>TCT/3
**Asn/5**	-	GTT/5	-	-
**Asp/11**	-	GTC/11	-	-
**Cys/4**	ACA/1	GCA/3	-	-
**Gln/8**	-	-	CTG/6	TTG/2
**Glu/15**	-	-	CTC/12	TTC/3
**Gly/22**	-	GCC/15	CC/3	TCC/4
**His/4**	-	GTG/4	-	-
**Ile/7**	AAT/6	-	-	TAT/1
**Leu/13**	AAG/5	-	CAG/4<br>CAA/2	TAG/1<br>TAA/1
**Lys/8**	-	-	CTT/6	TTT/2
**Phe/7**	-	GAA/7		-
**Pro/11**	AGG/5		CGG/4	TGG/2
**Ser/16**	AGA/4	GCT/5<br>GGA/1	CGA/4	TGA/2
**Thr/9**	AGT/4	-	CGT/3	TGT/2
**Trp/3**	-	-	CCA/3	-
**Tyr/4**	-	GTA/4	-	-
**Val/13**	AAC/8	-	CAC/3	TAC/2

Table S6. Metabolic pathways and gene distribution related to the synthesis of terpenoids in each group

Group	Pathway	out	Genes	enzyme	change
**MK-1 VS MK-2**	Terpenoid backbone biosynthesis	3	*Her005768;*	**iSPE** ；4-diphosphocytidyl-2-C-methyl-D-erythritol kinase	
			Her008335;Her011461	**FDPS**; farnesyl diphosphate synthase/**GGPPS**; geranylgeranyl pyrophosphate synthase	
**DK VS FB-A**	Terpenoid backbone biosynthesis	3	*Her005768;*	**iSPE** ；4-diphosphocytidyl-2-C-methyl-D-erythritol kinase	
			Her008335;Her011461	**FDPS**; farnesyl diphosphate synthase/**GGPPS**; geranylgeranyl pyrophosphate synthase	
	Ubiquinone and other terpenoid-quinone biosynthesis	2	*Her010901*	**ubiA**, 4-hydroxybenzoate polyprenyltransferase/ **COQ2**, 4-hydroxybenzoate polyprenyltransferase	
			Her001132	**ubiF**,3-demethoxyubiquinol 3-hydroxylase/**COQ7**, 3-demethoxyubiquinol 3-hydroxylase	
**DK VS FB-B**	Terpenoid backbone biosynthesis	3	*Her005768;*	**iSPE** ；4-diphosphocytidyl-2-C-methyl-D-erythritol kinase	up
			Her008335;Her011461	**FDPS**; farnesyl diphosphate synthase/**GGPPS**; geranylgeranyl pyrophosphate synthase	
	Ubiquinone and other terpenoid-quinone biosynthesis	3	*Her010901;Her009959*	**ubiA**, 4-hydroxybenzoate polyprenyltransferase/ **COQ2**, 4-hydroxybenzoate polyprenyltransferase	
			Her001132	**ubiF**,3-demethoxyubiquinol 3-hydroxylase/**COQ7**, 3-demethoxyubiquinol 3-hydroxylase	
**DK VS FB-C**	Terpenoid backbone biosynthesis	3	*Her005768;*	**iSPE** ；4-diphosphocytidyl-2-C-methyl-D-erythritol kinase	
			Her008335;Her011461	**FDPS**; farnesyl diphosphate synthase/**GGPPS**; geranylgeranyl pyrophosphate synthase	
	Ubiquinone and other terpenoid-quinone biosynthesis	3	*Her006816*	**TAT**,tyrosine aminotransferase/**ARO8**; aromatic amino acid aminotransferase I / 2-aminoadipate transaminase	
			Her009959;Her010901	**ubiF**,3-demethoxyubiquinol 3-hydroxylase/**COQ7**, 3-demethoxyubiquinol 3-hydroxylase	

Table S7. Identification of the putative gene clusters for terpene in *H. coralloide* genome

ID	source	start	end	strand
Her002335	terpene Chr02	3770782	3772380	-
Her002336	terpene Chr02	3773007	3774293	-
Her002337	terpene Chr02	3774779	3776006	-
Her002338	terpene Chr02	3777335	3778946	-
Her002339	terpene Chr02	3779922	3782626	-
Her002340	terpene Chr02	3783444	3785322	-
Her003785	terpene Chr04-1	700944	703377	+
Her003786	terpene Chr04-1	703806	705464	+
Her003787	terpene Chr04-1	705959	707873	+
Her003788	terpene Chr04-1	709199	710331	+
Her003789	terpene Chr04-1	710732	713935	-
Her003790	terpene Chr04-1	715347	716186	+
Her003791	terpene Chr04-1	716650	719582	-
Her003829	terpene Chr04-2	847822	848600	+
Her003830	terpene Chr04-2	848826	852210	-
Her003831	terpene Chr04-2	853261	854816	-
Her003832	terpene Chr04-2	856403	857546	+
Her003833	terpene Chr04-2	859232	860154	+
Her003834	terpene Chr04-2	860239	864362	-
Her003835	terpene Chr04-2	864588	865767	+
Her003836	terpene Chr04-2	866141	867270	-
Her005335	terpene Chr05-1	1874612	1876384	-
Her005336	terpene Chr05-1	1876823	1877882	+
Her005337	terpene Chr05-1	1879224	1882520	+
Her005338	terpene Chr05-1	1884547	1885693	-
Her005339	terpene Chr05-1	1888830	1889113	-
Her005340	terpene Chr05-1	1889146	1889524	-
Her005341	terpene Chr05-1	1894412	1895437	+
Her005430	terpene Chr05-2	2191114	2191650	-
Her005431	terpene Chr05-2	2193777	2194106	+
Her005432	terpene Chr05-2	2195390	2195927	-
Her005433	terpene Chr05-2	2197737	2199116	+
Her005434	terpene Chr05-2	2199405	2200822	-
Her005435	terpene Chr05-2	2201295	2202563	-
Her005436	terpene Chr05-2	2203145	2205339	-
Her005437	terpene Chr05-2	2206390	2208336	-
Her006719	terpene Chr06	3418024	3419917	-
Her006720	terpene Chr06	3420504	3421721	-
Her006721	terpene Chr06	3422072	3423089	-
Her006722	terpene Chr06	3423283	3424858	+
Her006723	terpene Chr06	3426242	3427399	+

Her006724	terpene	Chr06	3428788	3429389	+
Her006725	terpene	Chr06	3429881	3432073	-
Her006726	terpene	Chr06	3432893	3434190	+
Her006727	terpene	Chr06	3434712	3435485	+
Her006728	terpene	Chr06	3436222	3436862	+
Her006802	terpene	Chr07-1	116319	117786	+
Her006803	terpene	Chr07-1	120411	122161	-
Her006804	terpene	Chr07-1	125538	127207	+
Her006805	terpene	Chr07-1	127570	130132	-
Her006806	terpene	Chr07-1	130528	132325	-
Her006807	terpene	Chr07-1	132775	134807	+
Her007268	terpene	Chr07-2	1979414	1982469	+
Her007269	terpene	Chr07-2	1982889	1983433	-
Her007270	terpene	Chr07-2	1984569	1986882	-
Her007271	terpene	Chr07-2	1987399	1988560	+
Her007272	terpene	Chr07-2	1989002	1991786	-
Her007273	terpene	Chr07-2	1991968	1993739	+
Her007274	terpene	Chr07-2	1994089	1994573	-

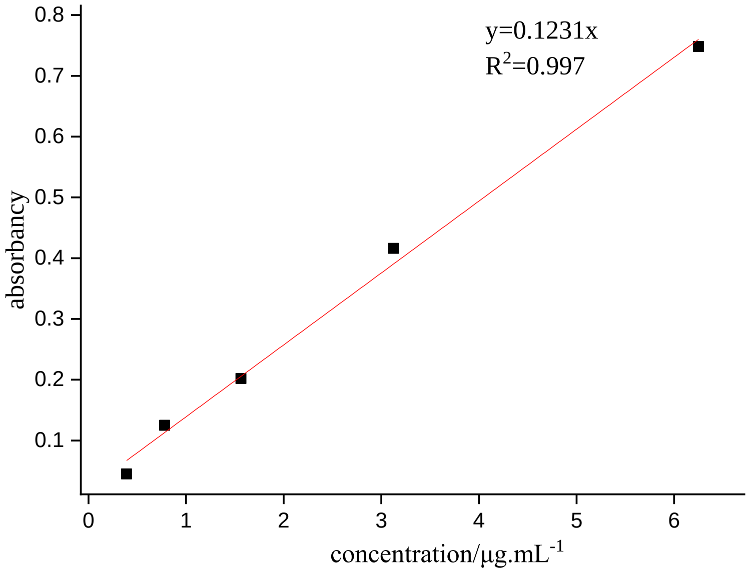

Figure S1. Calibration plot of scutellarin

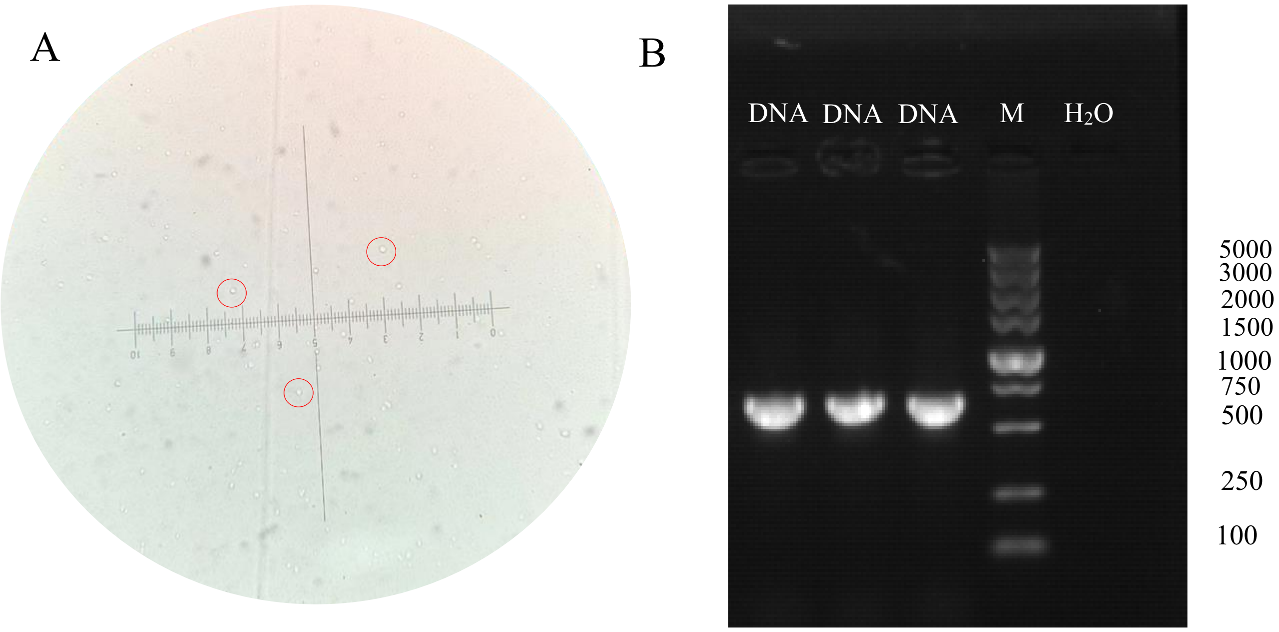

Figure S2. Protoplast mononuclearization results of DK. (A) Morphology of protoplasts DK under 40× microscope, the protoplasts are circled in red. (B) electropherogram of ITS-PCR.

A

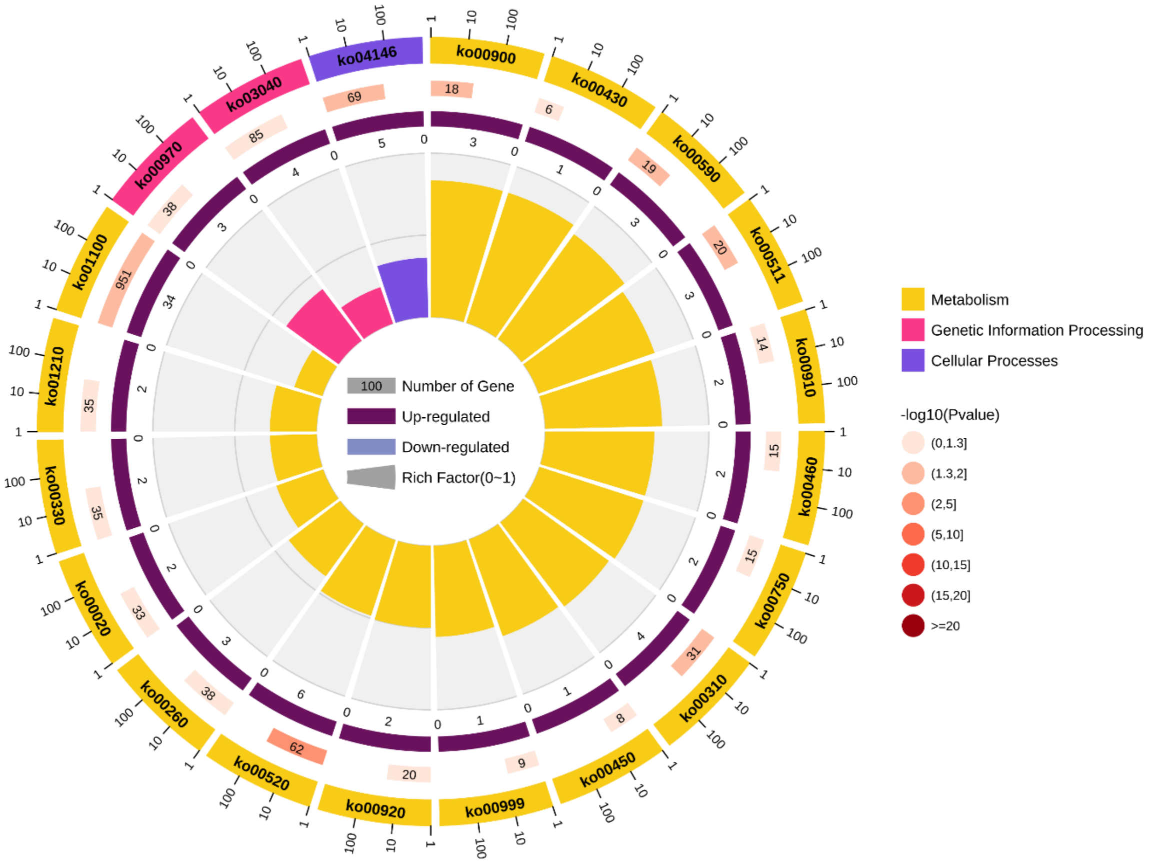
ko00900
ko00430
ko00590
ko00511
ko00910
ko00460
ko00750
ko00310
ko00450
ko00999
ko00920
ko00520
ko00260
ko00020
ko00330
ko01210
ko01100
ko00970
ko03040
ko04146
100 Number of Gene
Up-regulated
Down-regulated
Rich Factor(0~1)
Metabolism
Genetic Information Processing
Cellular Processes
-log10(Pvalue)
(0,1.3]
(1.3,2]
(2,5]
(5,10]
(10,15]
(15,20]
>=20

B

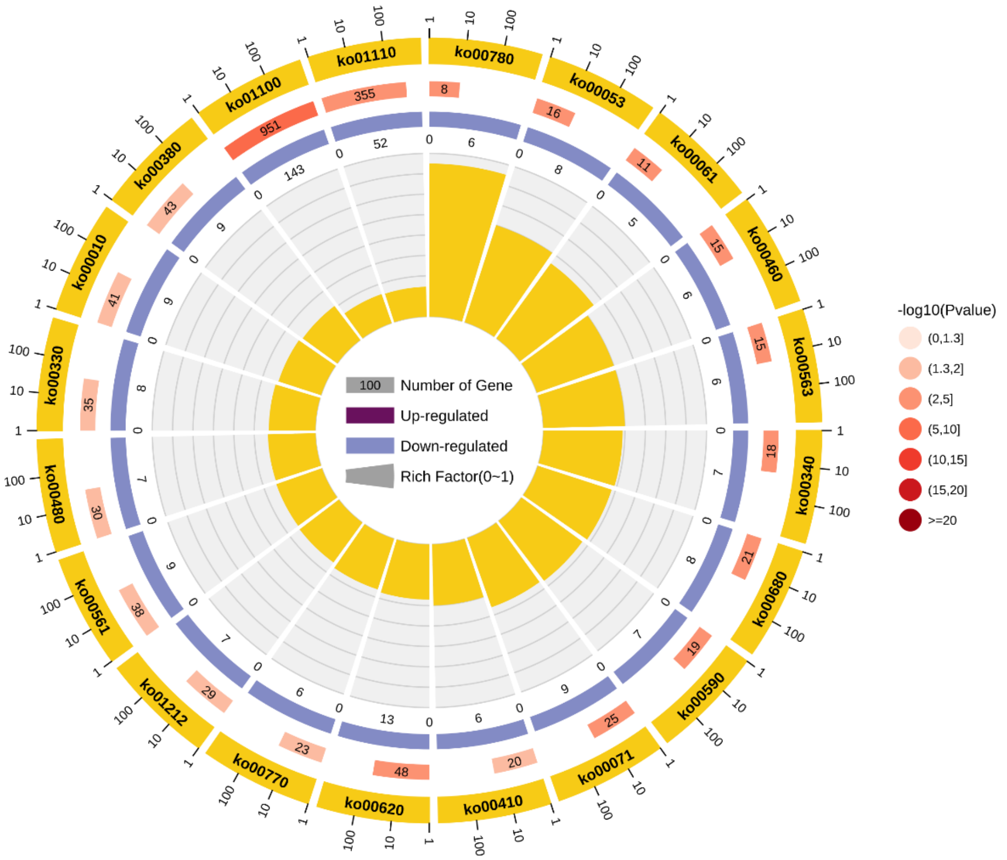
ko00780
ko00053
ko00061
ko00460
ko00563
ko00340
ko00680
ko00590
ko00071
ko00410
ko00620
ko00770
ko01212
ko00561
ko00480
ko00330
ko00010
ko00380
ko01100
ko01110
100 Number of Gene
Up-regulated
Down-regulated
Rich Factor(0~1)
-log10(Pvalue)
(0,1.3]
(1.3,2]
(2,5]
(5,10]
(10,15]
(15,20]
>=20

C

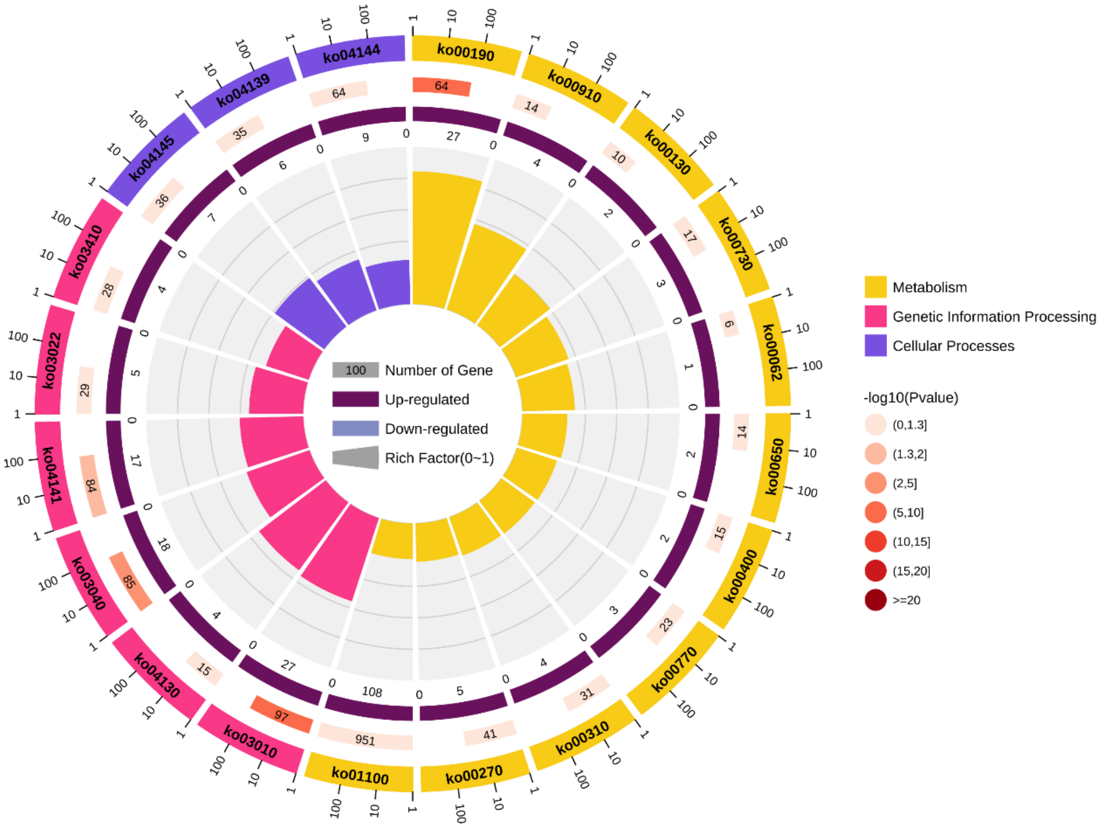
ko00190
ko00910
ko00130
ko00730
ko00062
ko00650
ko00400
ko00770
ko00310
ko00270
ko01100
ko03010
ko04130
ko03040
ko04141
ko03022
ko03410
ko04145
ko04139
ko04144
Number of Gene
Up-regulated
Down-regulated
Rich Factor(0~1)
Metabolism
Genetic Information Processing
Cellular Processes
-log10(Pvalue)
(0,1.3]
(1.3,2]
(2,5]
(5,10]
(10,15]
(15,20]
>=20

D

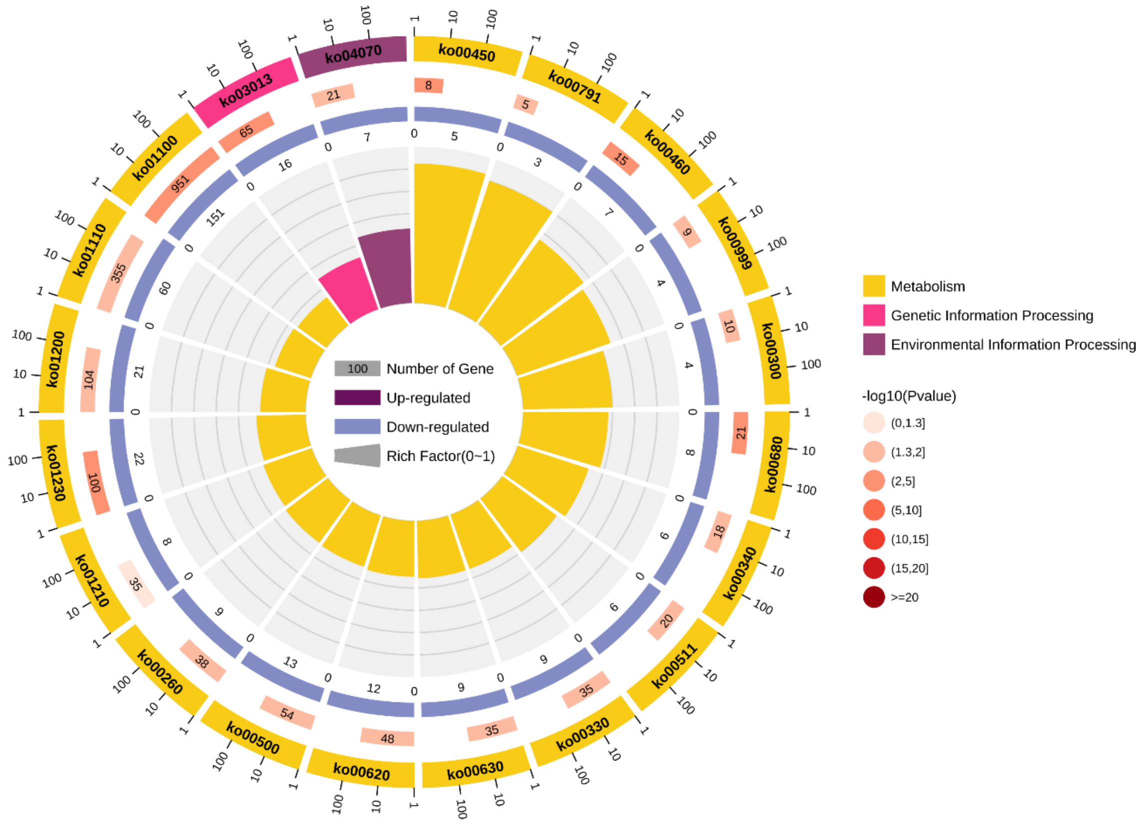
ko00450
ko00791
ko00460
ko00999
ko00300
ko00680
ko00340
ko00511
ko00330
ko00630
ko00620
ko00500
ko00260
ko01210
ko01230
ko01200
ko01110
ko01100
ko03013
ko04070
Number of Gene
Up-regulated
Down-regulated
Rich Factor(0~1)
Metabolism
Genetic Information Processing
Environmental Information Processing
-log10(Pvalue)
(0,1.3]
(1.3,2]
(2,5]
(5,10]
(10,15]
(15,20]
>=20

E

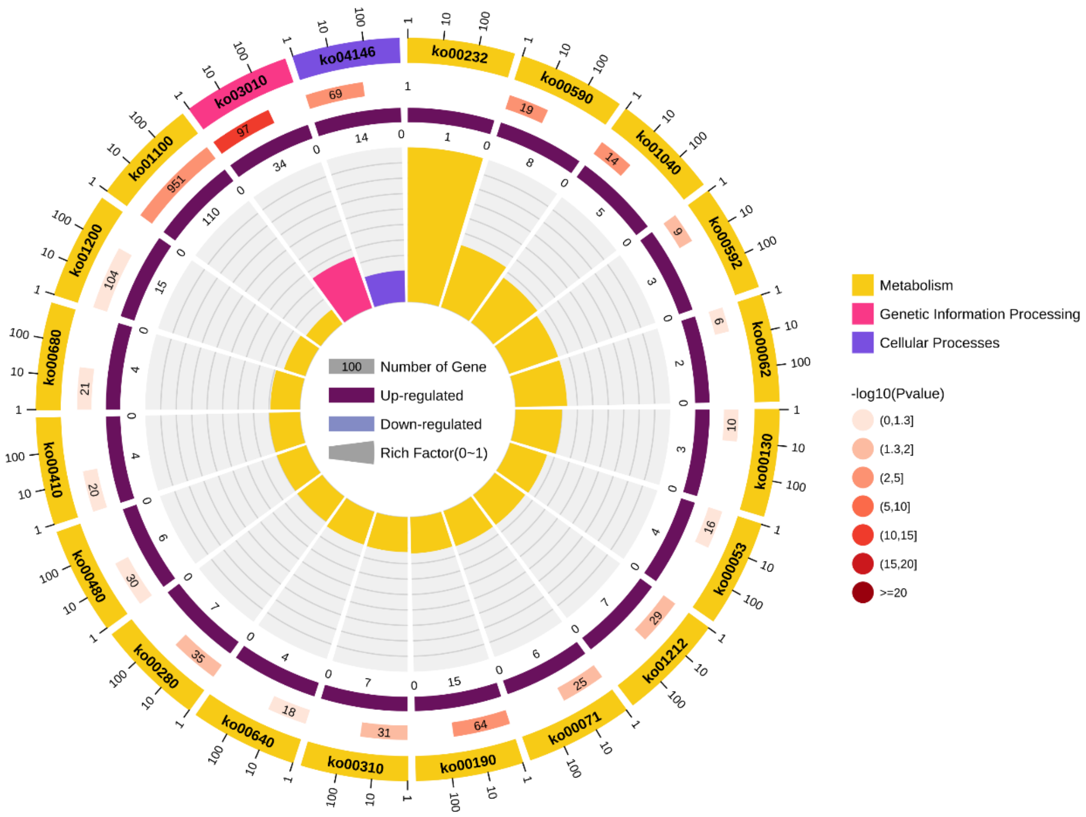
ko00232
ko00590
ko01040
ko00592
ko00062
ko00130
ko00053
ko01212
ko00071
ko00190
ko00310
ko00640
ko00280
ko00480
ko00410
ko00680
ko01200
ko01100
ko03010
ko04146
100 Number of Gene
Up-regulated
Down-regulated
Rich Factor(0~1)
Metabolism
Genetic Information Processing
Cellular Processes
-log10(Pvalue)
(0,1.3]
(1.3,2]
(2,5]
(5,10]
(10,15]
(15,20]
>=20

F

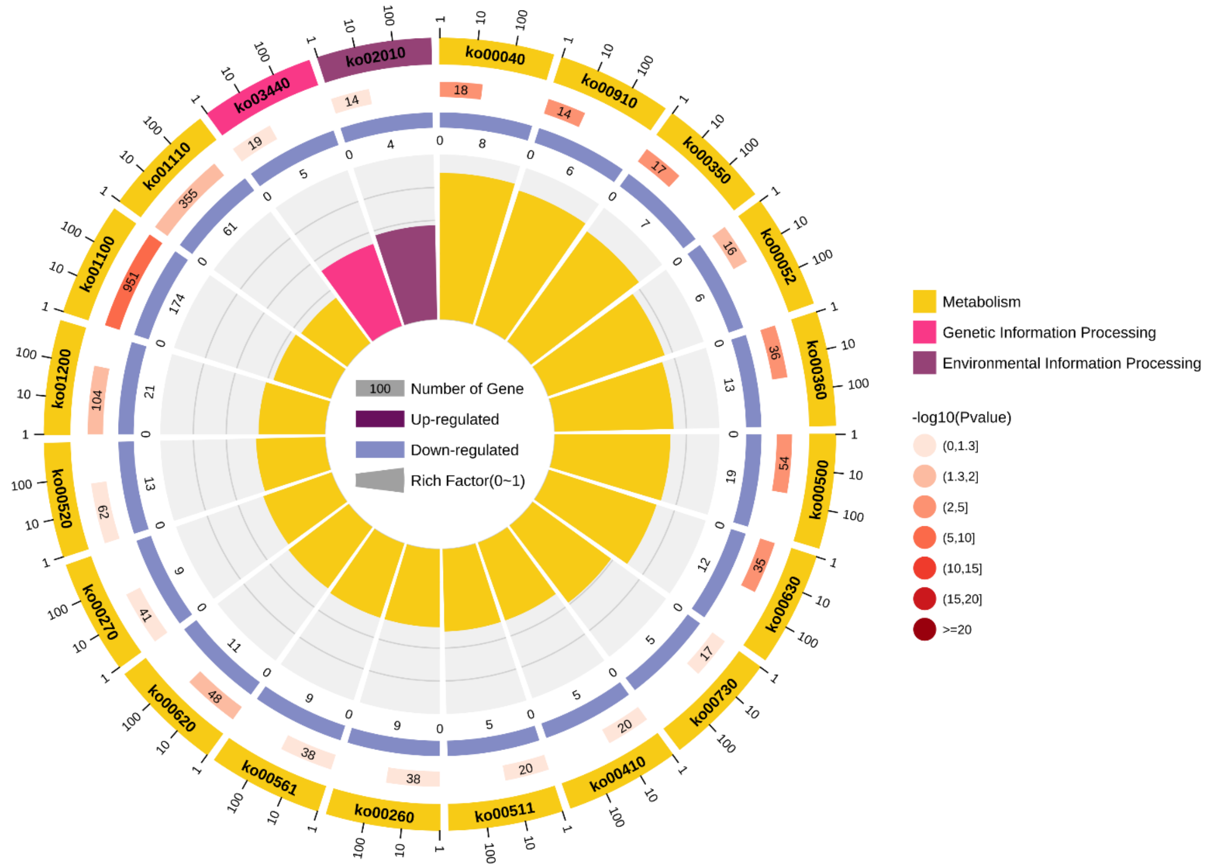
ko00040
ko00910
ko00350
ko00052
ko00360
ko00500
ko00630
ko00730
ko00410
ko00511
ko00260
ko00561
ko00620
ko00270
ko00520
ko01200
ko01100
ko01110
ko03440
ko02010
100 Number of Gene
Up-regulated
Down-regulated
Rich Factor(0~1)
Metabolism
Genetic Information Processing
Environmental Information Processing
-log10(Pvalue)
(0,1.3]
(1.3,2]
(2,5]
(5,10]
(10,15]
(15,20]
>=20

G

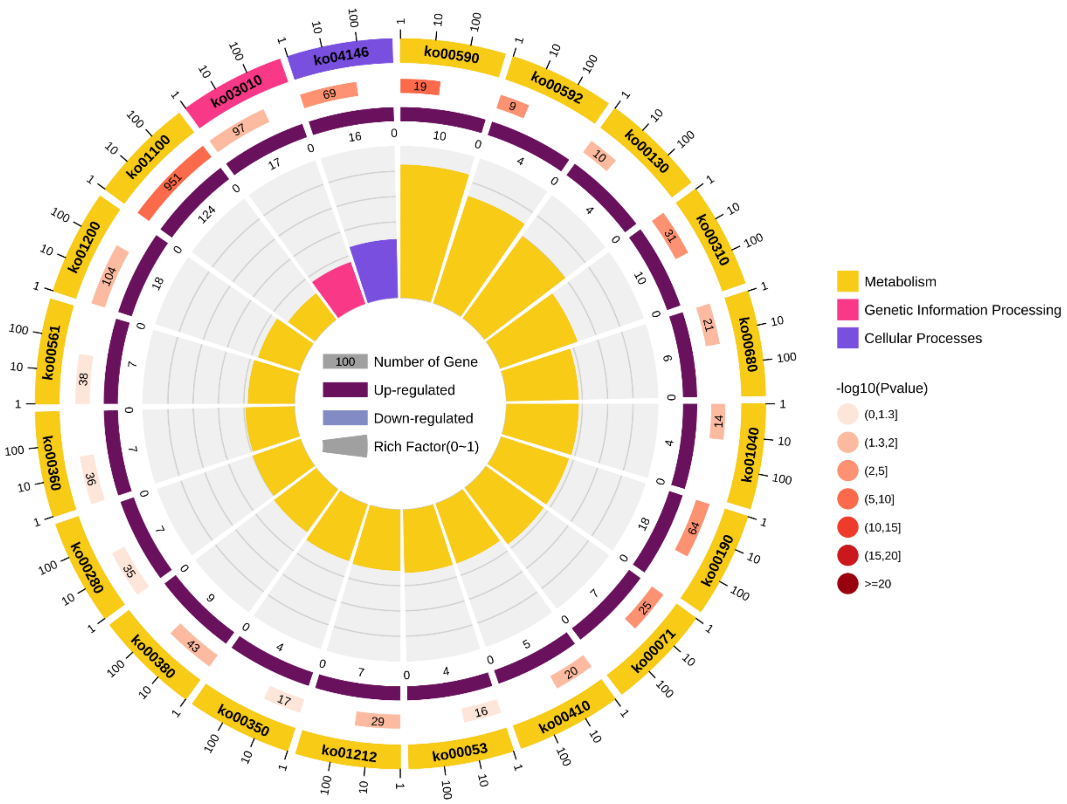
Metabolism
Genetic Information Processing
Cellular Processes
-log10(Pvalue)
(0,1.3]
(1.3,2]
(2,5]
(5,10]
(10,15]
(15,20]
>=20
Number of Gene
Up-regulated
Down-regulated
Rich Factor(0~1)

H

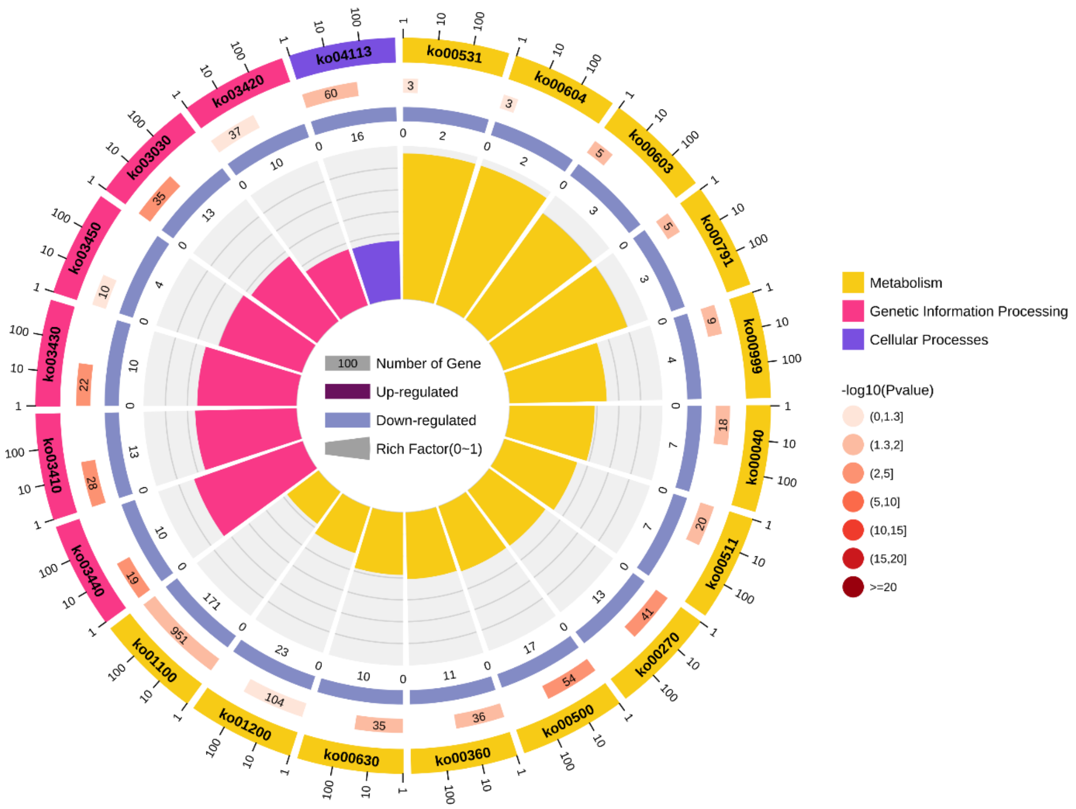
Metabolism
Genetic Information Processing
Cellular Processes
-log10(Pvalue)
(0,1.3]
(1.3,2]
(2,5]
(5,10]
(10,15]
(15,20]
>=20
Number of Gene
Up-regulated
Down-regulated
Rich Factor(0~1)

Figure S3. KEGG enrichment analysis of DEGs under different groups. (A-B) Up-regulated DEGs and down-regulated DEGs in MK-1 and MK-2 comparison groups. (C-D) Up-regulated DEGs and down-regulated DEGs in DK and FB-A comparison groups. (E-F) Up-regulated DEGs and down-regulated DEGs in DK and FB-B comparison groups. (G-H) Up-regulated DEGs and down-regulated DEGs in DK and FB-C comparison groups.

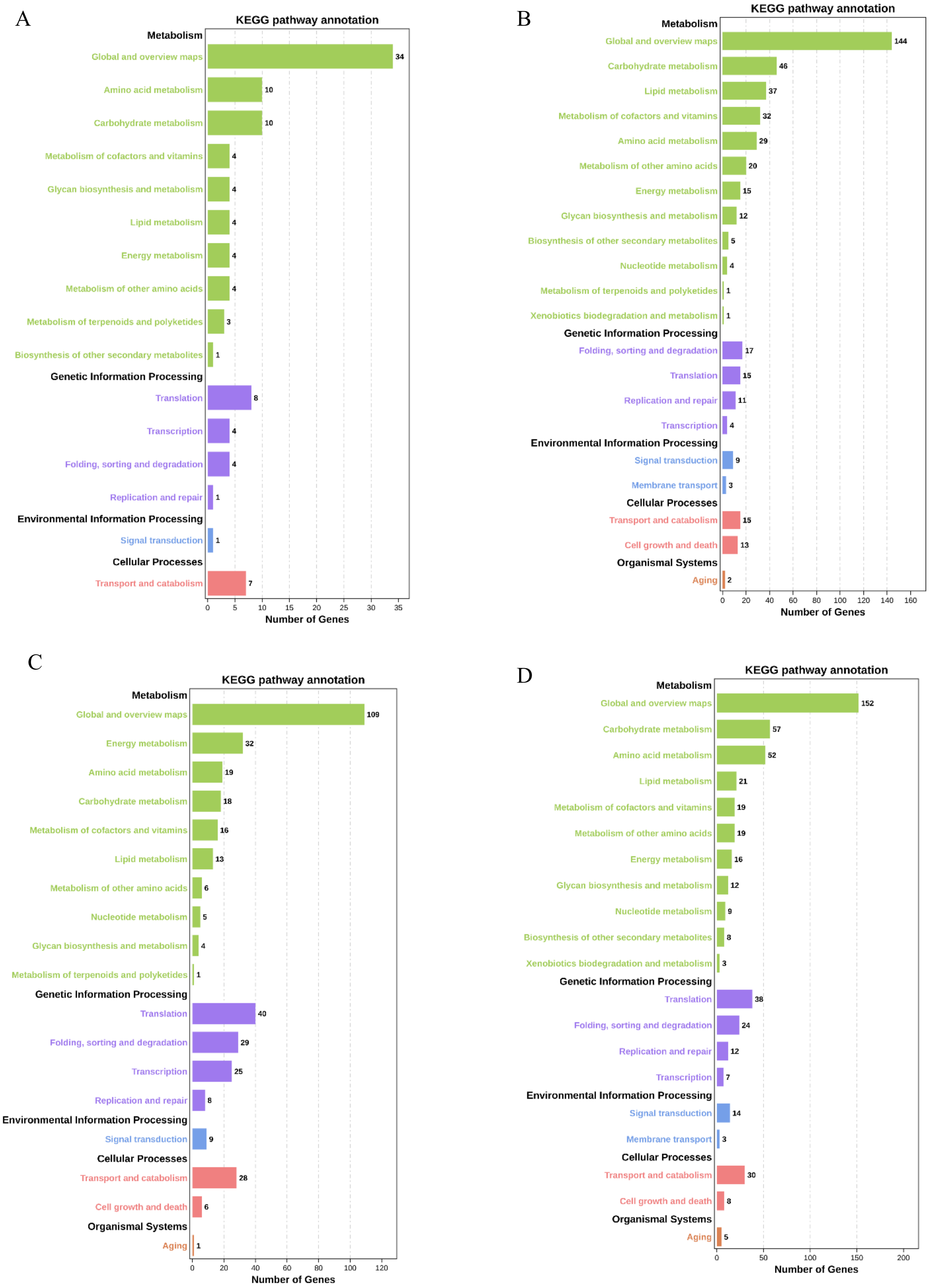
A
KEGG pathway annotation
Metabolism
Global and overview maps 34
Amino acid metabolism 10
Carbohydrate metabolism 10
Metabolism of cofactors and vitamins 4
Glycan biosynthesis and metabolism 4
Lipid metabolism 4
Energy metabolism 4
Metabolism of other amino acids 4
Metabolism of terpenoids and polyketides 3
Biosynthesis of other secondary metabolites 1
Genetic Information Processing
Translation 8
Transcription 4
Folding, sorting and degradation 4
Replication and repair 1
Environmental Information Processing
Signal transduction 1
Cellular Processes
Transport and catabolism 7
0 5 10 15 20 25 30 35
Number of Genes
B
KEGG pathway annotation
Metabolism
Global and overview maps 144
Carbohydrate metabolism 46
Lipid metabolism 37
Metabolism of cofactors and vitamins 32
Amino acid metabolism 29
Metabolism of other amino acids 20
Energy metabolism 15
Glycan biosynthesis and metabolism 12
Biosynthesis of other secondary metabolites 5
Nucleotide metabolism 4
Metabolism of terpenoids and polyketides 1
Xenobiotics biodegradation and metabolism 1
Genetic Information Processing
Folding, sorting and degradation 17
Translation 15
Replication and repair 11
Transcription 4
Environmental Information Processing
Signal transduction 9
Membrane transport 3
Cellular Processes
Transport and catabolism 15
Cell growth and death 13
Organismal Systems
Aging 2
0 20 40 60 80 100 120 140 160
Number of Genes
C
KEGG pathway annotation
Metabolism
Global and overview maps 109
Energy metabolism 32
Amino acid metabolism 19
Carbohydrate metabolism 18
Metabolism of cofactors and vitamins 16
Lipid metabolism 13
Metabolism of other amino acids 6
Nucleotide metabolism 5
Glycan biosynthesis and metabolism 4
Metabolism of terpenoids and polyketides 1
Genetic Information Processing
Translation 40
Folding, sorting and degradation 29
Transcription 25
Replication and repair 8
Environmental Information Processing
Signal transduction 9
Cellular Processes
Transport and catabolism 28
Cell growth and death 6
Organismal Systems
Aging 1
0 20 40 60 80 100 120
Number of Genes
D
KEGG pathway annotation
Metabolism
Global and overview maps 152
Carbohydrate metabolism 57
Amino acid metabolism 52
Lipid metabolism 21
Metabolism of cofactors and vitamins 19
Metabolism of other amino acids 19
Energy metabolism 16
Glycan biosynthesis and metabolism 12
Nucleotide metabolism 9
Biosynthesis of other secondary metabolites 8
Xenobiotics biodegradation and metabolism 3
Genetic Information Processing
Translation 38
Folding, sorting and degradation 24
Replication and repair 12
Transcription 7
Environmental Information Processing
Signal transduction 14
Membrane transport 3
Cellular Processes
Transport and catabolism 30
Cell growth and death 8
Organismal Systems
Aging 5
0 50 100 150 200
Number of Genes

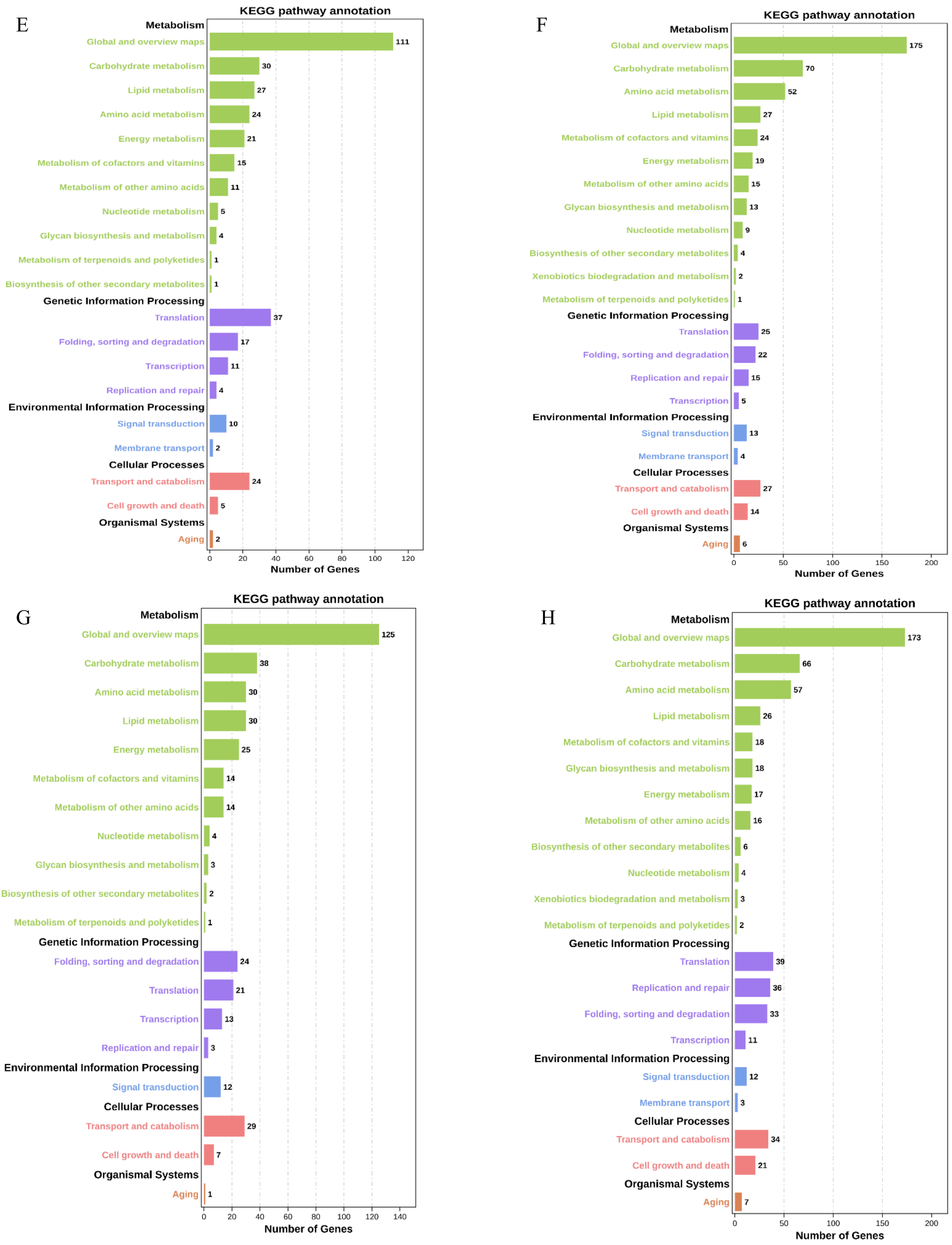
E
KEGG pathway annotation
Metabolism
Global and overview maps 111
Carbohydrate metabolism 30
Lipid metabolism 27
Amino acid metabolism 24
Energy metabolism 21
Metabolism of cofactors and vitamins 15
Metabolism of other amino acids 11
Nucleotide metabolism 5
Glycan biosynthesis and metabolism 4
Metabolism of terpenoids and polyketides 1
Biosynthesis of other secondary metabolites 1
Genetic Information Processing
Translation 37
Folding, sorting and degradation 17
Transcription 11
Replication and repair 4
Environmental Information Processing
Signal transduction 10
Membrane transport 2
Cellular Processes
Transport and catabolism 24
Cell growth and death 5
Organismal Systems
Aging 2
0 20 40 60 80 100 120
Number of Genes
F
KEGG pathway annotation
Metabolism
Global and overview maps 175
Carbohydrate metabolism 70
Amino acid metabolism 52
Lipid metabolism 27
Metabolism of cofactors and vitamins 24
Energy metabolism 19
Metabolism of other amino acids 15
Glycan biosynthesis and metabolism 13
Nucleotide metabolism 9
Biosynthesis of other secondary metabolites 4
Xenobiotics biodegradation and metabolism 2
Metabolism of terpenoids and polyketides 1
Genetic Information Processing
Translation 25
Folding, sorting and degradation 22
Replication and repair 15
Transcription 5
Environmental Information Processing
Signal transduction 13
Membrane transport 4
Cellular Processes
Transport and catabolism 27
Cell growth and death 14
Organismal Systems
Aging 6
0 50 100 150 200
Number of Genes
G
KEGG pathway annotation
Metabolism
Global and overview maps 125
Carbohydrate metabolism 38
Amino acid metabolism 30
Lipid metabolism 30
Energy metabolism 25
Metabolism of cofactors and vitamins 14
Metabolism of other amino acids 14
Nucleotide metabolism 4
Glycan biosynthesis and metabolism 3
Biosynthesis of other secondary metabolites 2
Metabolism of terpenoids and polyketides 1
Genetic Information Processing
Folding, sorting and degradation 24
Translation 21
Transcription 13
Replication and repair 3
Environmental Information Processing
Signal transduction 12
Cellular Processes
Transport and catabolism 29
Cell growth and death 7
Organismal Systems
Aging 1
0 20 40 60 80 100 120 140
Number of Genes
H
KEGG pathway annotation
Metabolism
Global and overview maps 173
Carbohydrate metabolism 66
Amino acid metabolism 57
Lipid metabolism 26
Metabolism of cofactors and vitamins 18
Glycan biosynthesis and metabolism 18
Energy metabolism 17
Metabolism of other amino acids 16
Biosynthesis of other secondary metabolites 6
Nucleotide metabolism 4
Xenobiotics biodegradation and metabolism 3
Metabolism of terpenoids and polyketides 2
Genetic Information Processing
Translation 39
Replication and repair 36
Folding, sorting and degradation 33
Transcription 11
Environmental Information Processing
Signal transduction 12
Membrane transport 3
Cellular Processes
Transport and catabolism 34
Cell growth and death 21
Organismal Systems
Aging 7
0 50 100 150 200
Number of Genes

Figure S4. KEGG pathway annotation of DEGs under different groups. (A-B) Up-regulated**(left)** DEGs and down-regulated DEGs **(right)** in MK-1 and MK-2 comparison groups. (C-D) Up-regulated DEGs **(left)** and down-regulated DEGs **(right)** in DK and FB-A comparison groups. (E-F) Up-regulated DEGs **(left)** and down-regulated DEGs **(right)** in DK and FB-B comparison groups. (G-H) Up-regulated DEGs **(left)**and down-regulated DEGs **(right)** in DK and FB-C comparison groups.

Data S1: The amino acid coding sequence of genes

>*Her002337*

MSLSRRASLALPSSPKSTHPSSFTLPDLVSHCTFPLTYHPAGDAVARDSLAWLLSFTGHLSARK
RGKMAHLLAGELTAHCYPTCPAFELRVVSDFMNYLFHLDDVSDGLIARDAGGLGRRVMHA
LECPDAYVPLEGLEGEEEISAARIARDFWSRCIRDARPGVQARFKESMRLFFDAVERQARDR
VAGVVPDLESYIDVRRDTSGCKPVFDLIEYSLGFELPAEVVAHPVVVALNQGANDLVTWSND
IFSYNKEQARDDTHNMVAIFMQHDGLSLQEAIDRVGELCKDTIDAFVANQARLPSWGPEVD
VMVQGYVRGLQDWIVGSLHWSFMTTRYFGDAGAEVKKSRVVQLMPKRPAGPWHRRLLSR
SLSLRR

>*Her003788*

MSFSAVQFTIPDLFALSTAFTDAVNPHWERASAESRAWVNGFQMFNDKRAAFFLSGQSELLV
SHAYPYAGYEEYRTVCDFVNLLFVMDEVSDRMNEKDALSTGEVYLKVMEDPTWDDGSKL
AQMTREFRERYTRRVQPNSNTFRRFLKLGKAYVECVAEEAGLRVRNEVLDLEKYTAIRRENS
AVRACFGVIEYVLGIDLPDEVFEDPVFQKIYFAGVDMVCWANDLYSFDMEQTKGLEGNNFL
TVLMRTRGMTLQEASDYTGAHYKELMDTFLTNEVQLRSFGPEVDKDVKLWIKSMQHWPV
GNLNWSFETPRYYGEARHEIRRTRVVTLRPLDKED

>*Her003832*

MPATVNRFFLPDLIALSGAFQDALNPHWKRAATESKQWVNSYNVFSDRRRAFFYKGQSELL
AAHAYPYAEYEEFRTVCDFINLLFVIDEMSDLQGCDEARKTGEIYLHVMRDPEWDDGSKLA
AMTRAFRERLTRTIKPGTFRRFLQHGQDYIDCVVDEAGLREKGNVLDLESYLVLRRENSAVR
TCFGLIPYVLGIDLPEEVYEDPAFKRIYFAGVDMVCWANDLYSYNMEQASGLAGNNFITVLM
QTKGLTLQQASDYTEEHYKSLMQTFVQESRNLRSFGPAVDADVKRYVEAMKHWPIGNVIW
SFETPRYFGPARETILKTREVILKPVEKPEDEAISLV

>*Her005338*

MPSLTRPMPRSFYIPDLLSLSTPFKGSTNPHYKKAAAESRAWINSYNVFTDRKRAFFVQGCN
ELLVSHTYPHAGYEEFRTCCDFVNLLFVVDEVSDDQNGEDARRTGEVYLNAMRDPEFDDGS
ALAKMTKEFRARLLRSTGPHAFARFLKHSEDYIDCVAKEAEYRERGEVLDMESFKHLRREN
SAIRLCFGLFEFTLGIDLPDEVFQDPVFSRLYWAAADMVCWANDVYSYNMEQAKGHTGNNI
VTVLMKATGLDLQGASDYIGVYYKDLMERYIADRARLPSFGPSLDRDVALYIRAMENWPIG
NLEWSFETNRYFGPMHDEVKRTRLVLLKPAAFEEDDQ

>*Her005433*

MASTASSSTITFPDFISYCDYPLRSNPSGADVAKASEQWLLDGAPLSEKKRKAFLGLKSGDLS
AMCYPDAEADRLRVVVDYMNYLFKLDDWSDEFEADEIHVMRDCVMGGLEDPVGFATEEP
VGLLTKSFFSRFIKTGGPGCRQRFIDTMELFFQAITQQAIDRINDDIPDLESYIALRRDTSGCKP
CFALIEYAAGIDLPDKVVNHPSMKILEDATNDLVSWSNDIFSFNVEQARGDTHNSVIIVMNEQ
GLDIQAAIDHVGSLCFATLDTFASVRSSLPSWGPDVDADVKKYVMGLQDWIIGSLHWSFMT
KRYFGKDGEEVKNTLKVALLPKKVAQQAQAQQAEVPQMQVEQVEVKAPQTQVESKPVVA
DAPGAASSNAPAGLLTAIWGMFGAAFRRATNSSAQTF

>*Her006723*

MVIANLVQDFLRRCNLVYPETDTDRLSSLIAACEAVSVSRGYITPEDDSFRKFIPAGVAMGYI
GYAHHPDENVQLYVTLYTAFLIYCDDMFENDILAVQEFNGRFMMGQPQKHWILDHLATFLR
DTPRYFNTVSTNLIITSTLNLVTALSFEYTLKGAQLEKSAVRYPGWGRIMCGAAEAYSLFIFSK
DVPLGNFLQSIPDIMIFTNYGNDVLSFYKEEMGAETINHVHSLAKCRNVHHSVTLREITEDAI
AAHHRALETLKPHKDAYDMYQRYSQGYVEFHTASKRYRLDELNL

>*Her006724*
MSALTEACMDVAVSRGYIVAEDNPLRKAIPVGAELAYIGYAHRLDEEVKLYIALYTAFLVHV
DDMFEHEPDAWMEFCGRLIKGQPQRHPILEHAASLFADAPVNIVASNVFLTSTLNFIIAPSLD
HELKGITLPASASRYPEWSRVYSGAAEAFSLFIFSRDIPLNVYLPVMPDLAFFLNYVK
>*Her010605*
MSLALGDLPEDTATKVAHIEVQDKARSFNEDKKHRGYTPEKLSRSEIKDLVENFLHQCGFVE
TTTDTLSDYAETCTSAWIARGLIAPEDNSVRSHIPLGSTIGCLAYGHHEDEETKLYISLHMTLI
AHLDDTFERYIDAMKEFNVRFMTGQPQKHACLDRFAAFLSTTSSHFNAIDADLIVTSTLNFIT
ALLLEHELKGIELEKSAISYLPYARDLDNATQAYALFIFPRRVPLKAYIHAVPDIMFFIKHTK
>*Her010608*
MFVLPMSSRLQWHPPDLPSLSMSSARKDLPEDCAVKIAGLRVQNEASELDEGKKDCNYTTE
KLPQAEIKDIVECFLRRCRFVKTTTGTISNYEETCTSASITRGYITLEDNSIRSSIPVGSAIGFLA
YGHLEDEVKLYASLYTALVTHLDDKFEHYINPMREFNRRFVAHQPQKHQFLDHVAAFLSTTS
NCFDAIAADLIVTSTLDFITFLLLEYDSKGVVLEKTAIPYVVYDRNINGIGRAYALFIFARNVPL
KSYIHALPHIMNFICHTK
>*Omp1*
MKYTSFALPDLASSCDYNLRFNKYHRSVSPETKKWFFRLSPASQADLTTYDAQRFTLLAAVC
YPDAGYPQLRVCSDFLAYLFYLDNLTDDMDDKSTRSVADLVLNSLNEPETFQTQYRIGKMTS
DYFKRIIQTSNDGTKKRFIDTMDSFFKSVDDQARDRLAGHIPDLESYIALRRETSGCKTCFSLI
EYANNLHIPDEVISHPHIEQMETAANDVVSFANDIYSFNIEQSKGDTHNMIPVLMHANPDMD
FLEAVSFVRDLTIKAMDRFNELRATLPSWGLDIDKDMKVYVNGLENWMVGILFWSFETERY
FGKSVRSVKATKTVNLLPSRA
>*Omp2*
MASTAPSKFILPDLVSHCSFDLHHNRHRKQITTETKKWLFKGDNLTGRKRDQYHGLKCGLLS
AMCYPNAAYPQLRVCNDFLTYLFHLDNLSDDMDNRGTTTTADVVLNSLYHPGYFQSARVG
KMTRDYWKRLISTASPGTQQRFIETFDFFFQSVTEQAHDRQAGVIPDLESYIALRRDTSGCKT
SFVLIEYANNLDIPDGVMDHPLIRSLGEAANDLVTWSNDIFSYNVEQAKGDTHNMIPVIMNE
HGLDLQSAVDYVGRLCQQSIDRFISDRAQLPSWGPEIDRQVAIYVDGLTDWIVGSLHWSFES
ERYFGKSGRQIKKSRVINLLPRRA
>*Omp3*
MAIENTIASAPASTPAKQLDTPDHFILPDLVSHCTFPLVYHSNGDAVAAQSVKWLDTNCPDLN
DKRRKALYGLQAGELTAYCYNTAPDQRLRVVSDFMNYLFHLDNISDGMMTKDTDALSDAV
MNALWFTEWYRPTKKSDYVQPDEELNAGKLARDFWHRCIQDAGPGCQARFKETLELFFEA
VNIQAKARDAGVIPDLESYIDVRRDTSGCKPCWALIEYGLGIDLPDYVAEDPIIKSLNQSTND
LVTWSNDIFSYNVEQSRGDTHNMIVILMLYHGHNLQSAIDYVGDLCRQTIDDFKENRKKIPS
WGPEVDDIVKQYVQGLQDWIVGSLHWSFMTTRYFGKQGQEVKKNRYVKLLPVGEEANKW
>*Omp4*
MSSAPTRFLLPDLLSACPLKGSVNPYYKEAGAESSAWINSYDIFTDRKRAFFVQGCNELLVA
HTYPYAGYEEFRTCCDFINVLFVLDEVSDEQSGSDARFTGEVFLNALRNPENDDTSKLSKISK
EFRARYFKRAGPRTAERFLQHCQDYIDCVTREAELRERGEVLDLPSFTALRRENSAIRVCFCL
FEYALGFDLPQEVFDDPTFMEMYWAAADLVCWANDVYSYNKEQAQGHGGNNIVTVLMKA
KDLDLQAACDYIGVYCEELMGRYLSAKARLPSWGPEVDAAVAQYVEASGHWVRGNLDWS
FETQRYFGAQHAEIKETRLVTLTPAIPEDFSDTGSESE
>*Omp5a*

MSPDPTRIVLPDFLAACPFESSTKNPHLKAAGAESSAWVNSHVVFNDRKRAAFMQDIYELLV
AYAFPWADYEDFRTMCDFINLLFVLDELSDDQNGKDAGYTGKLFMDAMRNIDNGDTSELTE
LCREFKARYSKRVSPQVNERFLQHLQSYTDCVAQEADLRERGEILDLESYVALRRENSAIRPC
FDLVEYIIDFDIPQEVIDHPVFSEMYWASVDLVCWSNDVYSYNVEQAKGHGGSNVVTVLMK
EKNLDLQAACDYVGVYYEELMDRYLSAKARLPSWGPEIDAAVGKYILAEAQFVRGNLDWS
FDSPRYFGPQHDQVKKTGIVTLTPAPKKFGSDSGSESE

>*Omp5b*

MSPAPSRIVLPDFFASCPFESSTINPHFKAAGAESSAWVNSHVVFNDRKRAALMQNSYELLVA
YAFPWASYEDFRTLCDFINLLFVFDEVSDDQNGKDAGYTSKIFMDAMRNIDNGDHSELTELC
KEFKARFSRRLSPQVNERFLQHLQSYTDCVAQEADLRERGEILDLESYVILRRENSAVRPCFD
LVEYIMDFDIPQEVLDHPVFSEMYWASVDLVCWSNDVYSYNVEQAKGHRGSNVVTVLMNE
KNLDLQAACDYVGVYYQELMDRYLSAKARLPSWGPEIDAAVGKYVLAEAQFVRGNLDWS
FDTPRYFGPQRDQIKKSRIVTLTPAPKKFGSDSGSESE

>*Omp6*

MIAKNSEIDRFYIPDTLANWPWPRHLNPAYPEAKKASAAWLRSFNAFNERSQKAFDLCDFNL
LASLAFPLADLYCLRSGCDLMNCFFIFDEYSDVADPQTVRQQADIIMDAIRNPHVPRPRGEFI
GGEAHRQFWERAMQGATPTAQRRFIDTYQQYTDAVVQQATDRADNHIRDVEGYFTVRRDT
IGAKPSFTLLEFTMDIPDEVMGHPVIKDLSLWCIDMLIIGNDLCSYNVEQAHGDDLHNLVTIV
MNQYNLDLPGAMEWIGKFHDDIADKFLDTFAKLPSWGPEIDPQIRRYVDGLGNWVRGNDS
WSFESWRYFRGKGPEIEKTRWVDLMPTEEATITPKYESDSNAAQPAQST

>*Omp7*

MPETFYLPDCLANWKWKRALNPNYPEVKAASSEWLRSFKAFPPKAQEAYDRCDFNLLASL
AYPLADKDGLRTGCDLMNMFFVFDEYSDVAHESEVQVQADIIMDALRNPHKPRPVGEWVG
GEVTRQFWELAIKTASPQSQKRFIETFDTYTKSVVQQAADRTQHYVRTVDEYLEVRRDTIGA
KPSFAILELTMDIPDEVIHHPTIERLAILAIDMILLGNDTASYNYEQARGDDNHNMVTIVMHQ
YKTDIQGALSWIEKYHKELEEEFMQLYNSLPKWGGQIDVDIARYVDGLGNWVRASDQWGF
ESERYFGTKAPEIQKTRWVTLMPKKRAEGVGPEIVDISEL

>*Omp9*

MSQILHLLWSKFSTSLPSTVTIGSDPQTLQLVHSPAPNVNANALEIYKIVDNFLSRCGIRLESTP
LDVEFYNECKKTLLSHYIGIHDSDKVSESWFKRYLSVGVIITTNAYGHLDNKLTKIYIALYTA
LATCFDDVFEKNVDHMSGFNERFMKALPQGDVFLDAFAKVLLDAPKYFGRLASNIIVTSTL
DFITSMSVDVLTKGMKFNQNLHKFAMACRNMSGIAYTYAPFIFPKEVPFAIYAQCLPDMRIYI
NHVNDVLSFYKEDKAGETENLASILGQVHPSMTKYQIVQGLADDAAEADLRVRTVLSQYQP
ALDAYNCFRQGYVSFHASSGRYRLDELFSFVEPEPIV

>*Omp10*

MTLPTEQVELSVCPVESSTHTTRDIMRNFLSQCQIPLQRGVPLDPTFHQECANVLIEDYLKPS
AAVTLENLPSLMSSFNPFLTLGVRMASTGYAHLTHTPTRVYVALFTALLVCLDDIFPENVELM
CGFNERFIKNETQGEPILDAVAGLLRSTSKYFSMLSSNLIVTSALNYVTSLSLDQGLHSIKLAE
HSRNFARLCRNMSGIPEAFAAFVFPPEVPFTAYIQCFPDLYTYANYVNDVLSFYKEDIAGETE
NLVSILAQTQPNSSRYQVLQQLADEAAAANANIRDILSDQKSILDAYDAFRVGFVQFHIDSPR
YRLAELFPCIDG

>*Cop1*

MSSLDATIHPVLNFEDKKIVLPDLVSHCNFKLRVSRHRKRITGETKRWLFKGDNLVGPARNK
YHGLKAGLLTAMTYPDAAYPQLRLCNDFLTYLFHIDNLSDDMDNRGTWSTANEVLNSLYHP

YTYHGQARVGRMTRDYWRRMILTASPGSQQRFIETFDFFFQSVTQQAIDRLTGEIPDLESYIA
LRRDTSGCKPCWALIEYANNLDLPDEVMDHPVVRSLGEAANDLVTWSNDIFSFNVEQSKGD
THNMIPVVMHQEGLDLQSAVDFVGEMCKSAIDRFIEDQNYLPSWGPKIDRDLAVYINGLAD
WIVGSLHWSFETERYFGKNGRQVKSSRVIDLLPRRSQ
>*Cop2*
MPSPAGALPKSFILPDLVNDCPFPLRVNPLCDEVGRLSEQWFLRHANYSPPRAVAFMALKAG
ELTAACYPDADAFHLRVSDDFMNFLFNADDWLDDFDIEDTYGLANCTVRALRDPVNFITDK
RAGLMTKSYFSRFLKTAGPRCTERFIQTLALYFESVVTQKQARNNGTLPDLESYITIRRNNSG
CKPCYALIEFCAGIDLPDEVINHPIIQSLEDASNDLIAWSNDIFSFNREQSRHDSFNMVSIVMH
QKGFALQEAVNFVGELCKKAMERFQADKRNLPSWGPEIDGEVAMYVDGLQNWIVGSLNW
SIDGTERYFGKDGPGIKKHRKVKLFPKRPLKTPAVRVLA
>*Cop3*
MSTPSSSLTTDESPASFILPDLVSHCPFPLRYHPKGDEVAKQTVHWLDSNCPDLTAKERKAMY
GLQAGELTGYCYPYTTPERLRVVADFLNYLFHLDNISDGMMTRETAVLADVVMNALWFPED
YRPTKGQAAEELNPGKLARDFWSRCIPDCGPGTQARFKETFGSFFEAVNIQARARDEGVIPD
LESYIDVRRDTSGCKPCWVLIEYALGIDLPDFVVEHPVIAALNQGTNDLVTWSNDIFSYNVE
QSKGDTHNMIIILMEHHGHTLQSAVDYVGSLCQQTINTFCENKQQLPSWGPEIDDMVAKYV
QGLEDWIVGSLHWSFQTRRYFGDEGQEIKQHRLVKLLTVAPPPPPPPPTPPPQSSDADTKKQK
VKAQDGKGPVSDEEVWALVRAEQSKGSILESLFGFLTTSLSRIFFGYFFAYSH
>*Cop4*
MRPTARQFTLPDLFSICPLQDATNPWYKQAAAESRAWINSYNIFTDRKRAFFIQGSNELLCSH
VYAYAGYEQFRTCCDFVNLLFVVDEISDDQNGQDARATGRIFVNAMRDAHWDDGSILAKIT
HEFRERFVRLAGPKTVRRFADLCESYTDCVAREAELRERNQVLGLNDFIALRRQNSAVLLCY
SLVEYILGIDLDDEVYEDPTFAKAYWAACDFVCWANDVYSYDMEQAKGHTGNNVVTVLM
KEKDLSLQEASDYIGRECEKQMRDYLEAKSQLLQSTDLPQEAVRYIEALGYWMVGNLVWS
FESQRYFGAQHERVKATHVVHLRPSSVLEASCDSDSDSDC
>*Cop6*
MPAALPYNVSRDNKWDIKKIIQDFFKRCDVPYQVIPYDTELWNACLKRAKEKGYPVEPDSP
MSLYRSFKVGVVITRTSYGHIQDYEILIWVATFTAFVTYADDAFQEDIQHLHSFARTFLQNEK
HEHPVLEAFAQFLRESSIRFSHFVANTVVSSALRFMMSIALEFEGQNVSVSTEAREYPGYIRIL
SGLSDIYALFAFPMDLPRSTYIQAFPEQIDYINGTNDLLSFYKEELDCETVNFISAAATSQQVS
KLEVLRNAAEKAAYSYDVVVNVLKPYPEALAAWKSFARGFCYFHTSSPRYRLGEMFHDFE
HDLVCKCASCTEI
>*Fompi1*
MTSTFYIPKTMANWPWQRAINPHYKEINAKSNAWLKTFKPFNEKSQIVFDKCAVEHLRTGC
DLMNLVFILDEYTDVEDANVVREMVDIVIDAIHNPEKPRPEGEVLLGEITRQFWALGIQTCTV
TARKHFEEAFTDYVISVYDQALNRATKSIHTVETYFKARRENIGIRPSYIPAVLGIDIADEAFY
HPMVVELAYLIAVLVVLDNDIYSYNKEQATGDDQYNIITIVMNQYGYTLDEAMKWTANCHE
EVEARFMKGMKELPYFGPEVDPQLQQFIQALALWPRANDCWSFESGRYFGSRGLEIQKTRT
VPTMPKVVDNRQSLRRENVVIPLID
>*SqtB*
MSTASSPSLVASEIDSPHHSRTSSPSPTLSPPTSFILPDLVSHCNFPLTYHPAGDEQAAASLAWM
LSFVP
HFTPKKVAAMNGLQAGELTAYCYHDCPPERLRVVDDFMNYLFHLDNISDGMMAKNTTQLA

DWVMNAFEWP
EKFQPTVNADGEVVEEIAAVKLARDYWSRCIQQAKPGVQQRFKSSMNMFFQAVEQQTNDR
DGQVVPDLES
YIDMRRDTSGCKPVFDLIEYALGFELPEEVVDHPVIKALNQDANDLVTWSNDVFSYNVEQA
RGDTHNMIC
IFMEHDGCTLQEAIDRVGGLCKQTIDAFVENKARVPSFAHLGPEVDAWTTGYVQGLQDWIV
GSLHWSFMT
KRYFQEAGAEVKKTRFVKLLPIEEGRHKHIPPIYASAMVAATA
>*Sh25180*
PRHINPHYQEVKKASAAWAESFGAFNPKAQHAYNACDFKRLRTGCDLMNMFFVFDEYSDV
SSPKDVIQQAAIIMDALRNPYAPRPDDEWVGGEVTRQFWKRAIKTATAGAQRRFIDAFESYT
QSVVQQAKDRHHGFIRDVDSYLEMRRETIGAKPSFVVLQMDMTLPDEVLAHPVIQQLSALS
TDMICLGNRRLIQILWTVQDICSYNVEQARGDDLHNIITIAMNQFDIDIAGAMDWVVKYHA
KLERKFLYLYNNGLPSWGKELDPQVERYVCGL
>*Sh64702*
MVRSPVSDKFCIPDTLASWPYPRILNPHYAEEKAASAAWTKGFGAFGPKAQDAFDRCDFKR
CRSGCDLMNLFFVIDEHSDTHGEETVRKMKDVVMDAIRNPHKPRPNDEWIGGEIARQFWER
AMCYASEISQRRFIDTFDEYLESVVDQAADRDSARIRDIESYINIRRNTIGAKPSFVIMEQGMD
IPDNVFENEVFQRLRMATIDMLCLGNDIVSYNIEQARGDDSHNIVRIVMNELDTDVPRAMD
WVAQRHTQLEREFFTALSELPTWGEPIDGWVKEYVYGLGNWVRANDQWSFESQRYFGTKG
MEIMKSRWLSVLPKVRPAEVGPQLVDQSLL
>*Sh73029*
MAVATSVATPVPTPAYSAGRAPAKEKKIYLPDTLAEWPWPRAINPHYAEAKEESQAWAASFN
AFSPKAQHAFNRCDFNLLASLAYPLATKHGCRSGCDLMNLFFVIDEYSDIAPVEEVRQQKDI
VMDALRNPHKPRPEGEWVGGEVARQFWALTITNASAQSQKHFIETFDEYLDSVVQQAEDRS
ESRIRDIQSYIDVRRNTIGAKPSFALLELDMDLPDEVLAHPTIQSLSLATIDMLCLGNDIVSYN
LEQARGDASHNIITIVMNELNLDVNGAMRWVGDFHKQLEKQFFEAFNNLPKWGNAELDAQ
IAVYCDGLGNWVRANDQWSFESERYFGARGLEIMETKTLAMMPIQRTEALGPQLVDDSIL
>*Sh128017*
MAAPESSAWVSSYNLFSDRKRTDFITGSNELLVSHTYPHADYDAFRTCCDFVNLLFVIDEISD
DQSGKAARRTGEVYLNAMRDPEWTDGSDLAKMTQQFRARFLRSVGPQSFRRFLRHSEDYI
DCVAKEAEYRERGQVLDMDSFKSLRRENSAIRLCFGLFEFTLGIDLPDSVFEDETFMKMYWA
SADMVCWANDVYSYNVEQAKGHSGNNIVTVLMAARDIDMQAASDYVGEYYAELMEEYM
TAKAELASKSFGSRDLDEDVWKYVNAMENWPIGNLEWSFKTNRYFGTLHDEVKRTRLVVI
KPRKVVV
>*Sh159379*
MAHPTTNPSHRLEQMESVREHIPRLQHFLGEIGYRHTTPPAPTLDFLHAHHHWIHHVLGPMT
SWTVAKLNALEDSSSTIFERAYPLSDAEMKFVLAKLTAIAIFLDDSLEDEETYDDIGNFAHRV
YLGEAQPTGVLTLYHQGIQELSKMHEGDAVFRGLAVAPWITFIDACMLEKRLLTFDSKLRVSP
RDLGYQRLRNSTDFTSLRAPKATPSEVEVSFPIFLRHKSGIGEAYAAAIFKSSRYQELPLSRFV
KSMPDMIYYIELVNDLMSFYKEQLAGETANLIHLQHQSWKGGQGTGPYGSWTLLDTFSRLC
DETRDAAFRVDELLRLDECEKIANGELRGEEVGLSPMDVTMAAQWREFRDGYVSWHLECQ
RYKLDFIKLSTFE
>*Armga1*

MSQRIFLPDTLANWQWPRHLNPHYAEVKKASAAWAKSFRAFQTKAQEAFDRCDFNLLASF
AYPLADEARLRSGCDLMNLFFVIDEYSDVSTEEEVRAQKDIVMDAIRNTEKPRPAGEWIGGE
VSRQFWDLAKKTASTQAQKRFIDTFDEYLESVVQQAADRNNSHVRGIESYLEVRRNTIGAK
PSFALLEFDMQLPDESHQSSGYQRNLRKSCIDMLCLGNDVVSYNLEQARDDDGHNIVTIAM
NELRTDVAGAMIWVDEYHKQLESRFMENFKKVPRWGGPIDLQVARYCDGLGNWVRANDQ
WSFESERYFGKKGPEIIQRRWITLMPKMVSEELGPQIVDGFHL
